# The FTO Mediated N6-Methyladenosine Modification of DDIT4 Regulation with Tumorigenesis and Metastasis in Prostate Cancer

**DOI:** 10.34133/research.0313

**Published:** 2024-02-21

**Authors:** Yue Zhao, Xin Hu, Haoran Yu, Huimin Sun, Lei Zhang, Chen Shao

**Affiliations:** ^1^ Department of Urology, Xiang’an Hospital of Xiamen University, Xiamen University, Xiamen, China.; ^2^ Department of Pathology, School of Basic Medicine, Binzhou Medical University, Yantai, China.; ^3^ State Key Laboratory of Urban Water Resource and Environment, Harbin Institute of Technology, Harbin, China.; ^4^ Department of Public healthy, Xiamen University, Xiamen, China.

## Abstract

The progression of numerous malignancies has been linked to N6-methyladenosine (m6A) alteration. However, the opposite trend of m6A levels in the development and metastasis of cancer has not been reported. This study aimed to evaluate the biological function and mechanism of fat mass and obesity-associated protein (FTO) in regulating m6A modification in prostate cancer development and epithelial–mesenchymal transition (EMT). An EMT model of LNCaP and PC-3 cells was established with transforming growth factor-β treatment, and FTO knockout cell line was established in prostate cancer cells using the CRISPR/Cas9 gene editing technology. The level of m6A modification in tumor tissues was higher than that in normal prostate tissues; m6A levels were decreased after EMT. FTO deletion increased m6A expression and enhanced PC-3 cell motility, invasion, and EMT both in vitro and in vivo. RNA sequencing and functional investigations suggested that DDIT4, a novel EMT target gene, plays a role in m6A-regulated EMT, which was recognized and stabilized by the m6A effector IGF2BP2/3. Decreased FTO expression was an independent indicator of worse survival, and the level of DDIT4 was considerably elevated in patients with bone metastasis. Thus, this study revealed that the m6A demethylase FTO can play different roles in prostate cancer as a regulator of EMT and an inhibitor of m6A modification. Moreover, DDIT4 can be suggested as a possible biomarker for prostate cancer metastasis prediction.

## Introduction

Prostate cancer is becoming increasingly common every year, and despite its low malignancy, the failure of antiandrogenic treatment and occurrence of metastasis still lead to high mortality [1–3]. The biggest concern with prostate cancer is that it has a tendency to metastasize. This propensity stems from local infiltration, extravasation, and distal migration from the primary site, subsequently progressing to the specific establishment of endothelial attachment, transit, and secondary site metastasis [4]. Epithelial cells undergo epithelial–mesenchymal transition (EMT), which transforms them into mesenchymal cells that participate in distant tumor metastasis. EMT is considered the early stage of tumor metastasis and has a complex regulatory process that usually involves the dissolution of cell–cell junctions and loss of original cell polarity. Tumor cells with the EMT phenotype are highly mobile to infiltrate locally and reach target organs via secondary metastasis by invading blood vessels and lymphatic vessels. Being a hormone-dependent tumor, prostate cancer requires the androgen and androgen receptor (AR) signal axis to maintain normal function. Androgen has been demonstrated to have an impact on EMT by inhibiting the expression of E-cadherin [5,6]. However, there is a need for additional studies on the mechanism of how EMT in prostate cancer is controlled by the interplay of AR with EMT-related transcription factor expression.

N6-methyladenosine (m6A), the most widespread modification on mRNA, is involved in the regulation of almost all malignant tumors [7]. Methylated RNA immunoprecipitation and sequencing (MeRIP-seq), widely used to analyze m6A sites, revealed that m6A regions are contained in approximately 100-nt sequences [8]. The m6A methyltransferase complex controls dynamic and reversible biological processes that determine m6A levels. In this context, METTL3 was the first discovered methyltransferase. It is the core subunit of a “writer” complex that methylates RNA at the adenosine of m6A [9,10]. m6A can control RNA expression through a reversible process aided by “writers”, blocked by “erasers”, and performed by “readers”. Fat mass and obesity-associated protein (FTO) was the first eukaryotic m6A demethyltransferase to be discovered, and its involvement in adipogenesis and cancer has been linked to its m6A demethylase function [11]. Moreover, FTO has been demonstrated to play a role in cancer, operating as an oncoprotein in leukemia and participating in critical biological processes [12,13]. Likewise, Zhen et al. [14] showed that patients with pancreatic ductal carcinoma who expressed FTO exhibited a poor prognosis and that suppressing FTO expression prevented cell growth. However, according to Jeschke et al. [15], widespread FTO down-regulation in epithelial malignancies has been linked to poorer clinical outcomes, increased invasion, and metastasis. Of note, the function of m6A enzymes might vary or even be opposite in certain malignancies. Conflicting findings have revealed that cancer subtypes and tissue origin played a tumor suppressor/activator role [16]. These findings collectively imply that FTO expression may control cancer development.

By regulating EMT-related molecules, m6A can indirectly increase or decrease the intensity of EMT. Down-regulated FTO levels in prostate and breast cancers are associated with prognosis, but the exact mechanism is unclear [16,17]. Using research results obtained by Lin et al. [18], METTL3-modified Snail mRNA was suggested to exert a heterogeneous influence on various tumor models. Moreover, m6A levels were significantly increased in cervical cancer and liver cancer cells undergoing EMT. In the present study, an EMT model of prostate cancer was established from the perspective of altered m6A RNA methylation levels to identify m6A regulators that affect prostate cancer progression. The study results revealed down-regulated m6A methylation levels in prostate cancer cells undergoing EMT, with FTO as the regulator. Using sequencing technology combined with MeRIP-quantitative polymerase chain reaction (qPCR) analysis, an EMT target gene regulated by the m6A level was identified. The study revealed a novel regulatory network of prostate cancer metastasis, thereby laying a theoretical foundation for the molecular diagnosis and individualized treatment of metastatic prostate cancer.

## Results

### m6A level is up-regulated in prostate cancer

**Fig. 1.**
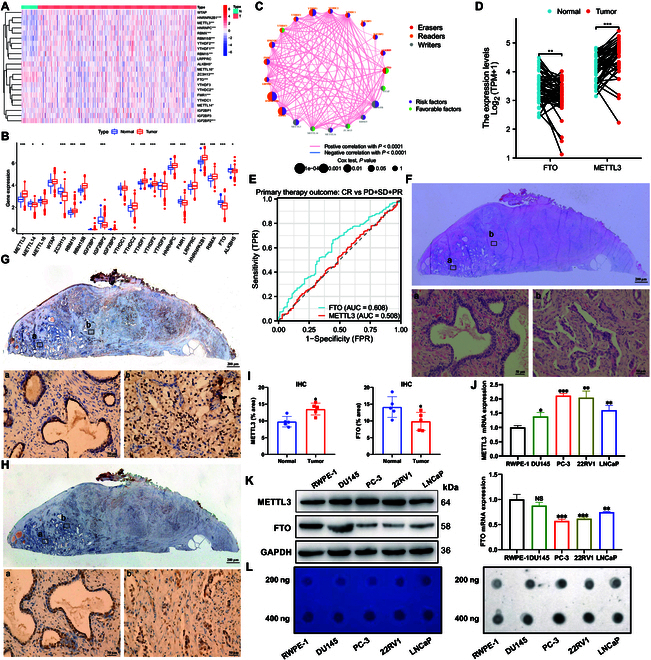
METTL3 and FTO regulate the m6A level in prostate cancer. (A) Heatmap of differentially expressed m6A regulators. (B) RNA expression of m6A regulators in tumor and normal tissues. (C) Protein–protein interactions among m6A regulators. The circle size represents the effect of each regulator on the prognosis. Green dots in the circle, risk factors of prognosis; large dots in the circle, protective factors of prognosis. (D) Paired expression level of METTL3 and FTO in prostate cancer and tumor-adjacent tissues. (E) ROC curve of FTO and METTL3 predicted the primary therapy outcome. (F) H&E staining results of prostate cancer and tumor-adjacent tissues. Representative images of tumor-adjacent tissues are shown in (a) and those of tumor tissues are shown in (b). (G) IHC (METTL3)-stained paraffin-embedded sections obtained from patients with prostate cancer. Representative images of tumor-adjacent tissues are shown in (a) and those of tumor tissues are shown in (b). (H) IHC (FTO)-stained paraffin-embedded sections obtained from patients with prostate cancer. Representative images of tumor-adjacent tissues are shown in (a) and those of tumor tissues are shown in (b). (I) Quantitative IHC analysis of METTL3 and FTO. (J) RNA expression of METTL3 and FTO was measured using qRT-PCR. (K) Protein expression of METTL3 and FTO was measured using western blot. (L) Global m6A RNA level was measured via m6A dot blot assays. Methylene blue stain served as the loading control.

To identify enzymes that regulate m6A level in prostate cancer, differentially expressed 6 methyltransferases and 2 demethyltransferases were identified from the The Cancer Genome Atlas (TCGA) database (Fig. 1A and B). These differentially expressed genes (DEGs) were divided into biological process, cellular component, and molecular function terms to better understand their biological functions. The top 3 identified biological process (*P* < 0.05) terms are mainly involved in metabolic process regulation and mRNA and RNA stability (Fig. S1A). The results of Kyoto Encyclopedia of Genes and Genomes (KEGG) enrichment analysis showed that the above DEGs were highly enriched in the spliceosome, interleukin-17, and Amp-activated protein kinase pathways (Fig. S1B). Protein–protein interactions among m6A regulators and their prognostic significance in prostate cancer are depicted in Fig. 1C. A previous study revealed that tumors with poor FTO expression displayed low “writer” expression (including KIAA1429, METTL14, RBM15, RBM15B, ZC3H13, RBMX, and CBLL1) but high METTL3 expression [19]. In the matched clinical samples, the FTO level was down-regulated and METTL3 was up-regulated in most prostate cancer samples (Fig. 1D). Receiver operating characteristic (ROC) curve analysis showed that compared with METTL3, FTO was a better predictor of primary therapy outcome (Fig. 1E). Normal prostate and prostate cancer tissues were differentiated using hematoxylin and eosin (H&E) staining (Fig. 1F). Immunohistochemistry (IHC) results showed that METTL3 was up-regulated in prostate cancer, whereas FTO was down-regulated (Fig. 1G to I). The RNA and protein levels of METTL3 and FTO in RWPE-1, LNCaP, PC-3, 22RV1, and DU145 cells were assessed, and the results showed that METTL3 expression was up-regulated while FTO expression was down-regulated in prostate cancer cells (Fig. 1J and K). Dot-blot assays performed to examine the m6A level in RWPE-1, LNCaP, PC-3, 22RV1, and DU145 cells revealed that the m6A level was increased in prostate cancer cells (Fig. 1L). The above results suggested that changes in m6A levels in prostate cancer are consistent with the trends of METTL3 and FTO expression.

### m6A level is regulated by FTO in TGF-β-treated prostate cancer cells

**Fig. 2.**
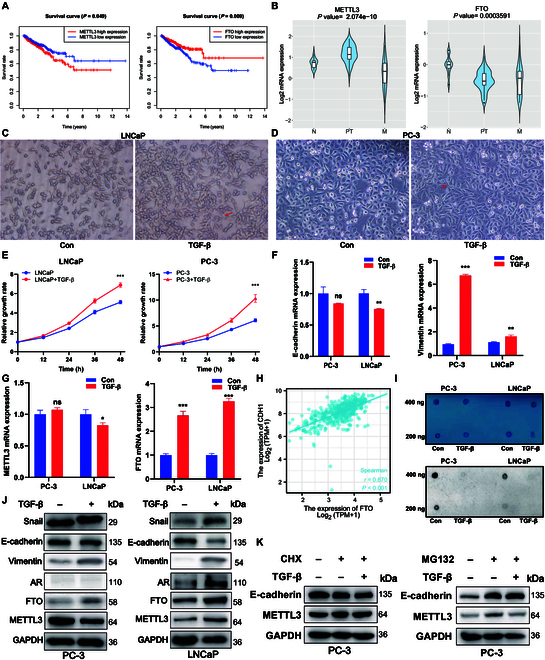
Construction of an EMT model for prostate cancer to verify change in the m6A level. (A) Kaplan–Meier survival curves of DFS based on METTL3 and FTO. (B) Expression level of METTL3 and FTO in normal tissues, primary tumors, and metastatic tissues. (C) Phenotypic changes in LNCaP prostate cancer cells treated with TGF-β. (D) Phenotypic changes in PC-3 prostate cancer cells treated with TGF-β. (E) Cell proliferation capacity changes in prostate cancer cells treated with TGF-β. (F) mRNA level of vimentin and CDH1 was measured using qRT-PCR. (G) mRNA level of METTL3 and FTO was measured using qRT-PCR. (H) Correlation curve of CDH1 and FTO expression levels. (I) Global m6A RNA level was measured via m6A dot blot assays. (J) Protein level of EMT markers and m6A regulators was measured using western blot analysis. (K) PC-3 cells were pretreated with CHX or MG-132 for 6 h and then further treated with or without 10 ng/ml TGF-β for 48 h. METTL3 expression was then detected using western blot analysis.

METTL3 and FTO are closely related to the prognoses of multiple tumors, but their regulatory processes and mechanisms have obvious differences. Thus, clinicopathological factors were analyzed and Kaplan–Meier survival analysis was performed based on TCGA data. The results showed that prostate cancer patients having higher METTL3 expression and lower FTO expression (Fig. 2A) exhibited reduced disease-free survival (DFS). However, the change in expression levels was not statistically for predicting overall survival (OS) (Fig. S1C and D). METTL3 expression was only closely related to Gleason score (Table 1), whereas FTO expression was strongly correlated with the first treatment outcome and progression-free interval (PFS) (Table 2). Next, it was found that METTL3 expression was decreased and FTO expression was increased in the metastatic prostate cancer samples (Fig. 2B). LNCaP and PC-3 cells treated with transforming growth factor-β (TGF-β) for 48 h became dispersed and assumed the fibroblast-like morphology of mesenchymal cells (Fig. 2C and D). TGF-β treatment dramatically enhanced the cell proliferation capacity of both LNCaP and PC-3 cells in vitro (Fig. 2E). Moreover, quantitative reverse transcription PCR (qRT-PCR) in LNCaP cells revealed the up-regulation of vimentin and FTO mRNA and down-regulation of CDH1 and METTL3 mRNA (Fig. 2F and G). Moreover, CDH1 was associated with FTO expression (Fig. 2H). Therefore, the insignificant decrease in CDH1 expression level may be related to the substantial increase in FTO expression level after EMT. Dot-blot assays used to examine the m6A level in the EMT models of PC-3 and LNCaP cells showed that the m6A level was decreased after treatment with TGF-β (Fig. 2I). Western blot analysis further supported the finding of TGF-β-induced alterations in EMT marker expression (Fig. 2J). These findings collectively demonstrated that TGF-β-treated cancer cells undergo EMT processes. However, the expression level of METTL3 was only reduced in PC-3 cells treated with TGF-β and not in LNCaP cells where it increased. Therefore, the pretreatment of PC-3 cells with cycloheximide (CHX) or MG-132 for 6 h, followed by treatment with or without TGF-β for 48 h, revealed METTL3 expression, which was detected using western blot analysis. Thus, MG-132, but not CHX, prevented TGF-β-induced METTL3 expression in PC-3 cells (Fig. 2K).

**Table 1.** Relationship between the expression level of METTL3 and clinicopathological factors

Characteristic	Low expression of METTL3	High expression of METTL3	*P* value
*n*	249	250	
T stage, *n* (%)			0.953
T2	94 (19.1%)	95 (19.3%)	
T3	146 (29.7%)	146 (29.7%)	
T4	6 (1.2%)	5 (1%)	
N stage, *n* (%)			0.342
N0	177 (41.5%)	170 (39.9%)	
N1	35 (8.2%)	44 (10.3%)	
M stage, *n* (%)			0.619
M0	231 (50.4%)	224 (48.9%)	
M1	1 (0.2%)	2 (0.4%)	
Primary therapy outcome, *n* (%)			0.073
PD	16 (3.7%)	12 (2.7%)	
SD	8 (1.8%)	21 (4.8%)	
PR	20 (4.6%)	20 (4.6%)	
CR	178 (40.6%)	163 (37.2%)	
Age, *n* (%)			0.555
<=60	108 (21.6%)	116 (23.2%)	
>60	141 (28.3%)	134 (26.9%)	
Residual tumor, *n* (%)			0.163
R0	166 (35.5%)	149 (31.8%)	
R1	68 (14.5%)	80 (17.1%)	
R2	1 (0.2%)	4 (0.9%)	
PSA (ng/ml), *n* (%)			0.961
<4	210 (47.5%)	205 (46.4%)	
>=4	13 (2.9%)	14 (3.2%)	
Gleason score, *n* (%)			< 0.001
6	21 (4.2%)	25 (5%)	
7	146 (29.3%)	101 (20.2%)	
8	21 (4.2%)	43 (8.6%)	
9	59 (11.8%)	79 (15.8%)	
10	2 (0.4%)	2 (0.4%)	
OS event, *n* (%)			0.751
Alive	245 (49.1%)	244 (48.9%)	
Dead	4 (0.8%)	6 (1.2%)	
DSS event, *n* (%)			0.372
Yes	247 (49.7%)	245 (49.3%)	
No	1 (0.2%)	4 (0.8%)	
PFI event, *n* (%)			0.216
Yes	208 (41.7%)	197 (39.5%)	
No	41 (8.2%)	53 (10.6%)	
Age, median (IQR)	61 (57, 66)	62 (56, 66)	0.138

**Table 2.** Relationship between the expression level of FTO and clinicopathological factors

Characteristic	Low expression of FTO	High expression of FTO	*P* value
*n*	249	250	
T stage, *n* (%)			0.105
T2	96 (19.5%)	93 (18.9%)	
T3	147 (29.9%)	145 (29.5%)	
T4	2 (0.4%)	9 (1.8%)	
N stage, *n* (%)			0.789
N0	171 (40.1%)	176 (41.3%)	
N1	37 (8.7%)	42 (9.9%)	
M stage, *n* (%)			1.000
M0	228 (49.8%)	227 (49.6%)	
M1	2 (0.4%)	1 (0.2%)	
Primary therapy outcome, *n* (%)			0.014
PD	14 (3.2%)	14 (3.2%)	
SD	18 (4.1%)	11 (2.5%)	
PR	27 (6.2%)	13 (3%)	
CR	150 (34.2%)	191 (43.6%)	
Age, *n* (%)			0.095
<=60	102 (20.4%)	122 (24.4%)	
>60	147 (29.5%)	128 (25.7%)	
Residual tumor, *n* (%)			0.140
R0	149 (31.8%)	166 (35.5%)	
R1	84 (17.9%)	64 (13.7%)	
R2	2 (0.4%)	3 (0.6%)	
PSA (ng/ml), *n* (%)			0.501
<4	205 (46.4%)	210 (47.5%)	
>=4	11 (2.5%)	16 (3.6%)	
Gleason score, *n* (%)			0.867
6	21 (4.2%)	25 (5%)	
7	120 (24%)	127 (25.5%)	
8	32 (6.4%)	32 (6.4%)	
9	74 (14.8%)	64 (12.8%)	
10	2 (0.4%)	2 (0.4%)	
OS event, *n* (%)			0.063
Alive	241 (48.3%)	248 (49.7%)	
Dead	8 (1.6%)	2 (0.4%)	
DSS event, *n* (%)			0.214
Yes	243 (48.9%)	249 (50.1%)	
No	4 (0.8%)	1 (0.2%)	
PFI event, *n* (%)			0.049
Yes	193 (38.7%)	212 (42.5%)	
No	56 (11.2%)	38 (7.6%)	
Age, median (IQR)	62 (57, 66)	61 (56, 66)	0.088

### FTO knockout enhances m6A level in prostate cancer cells

**Fig. 3.**
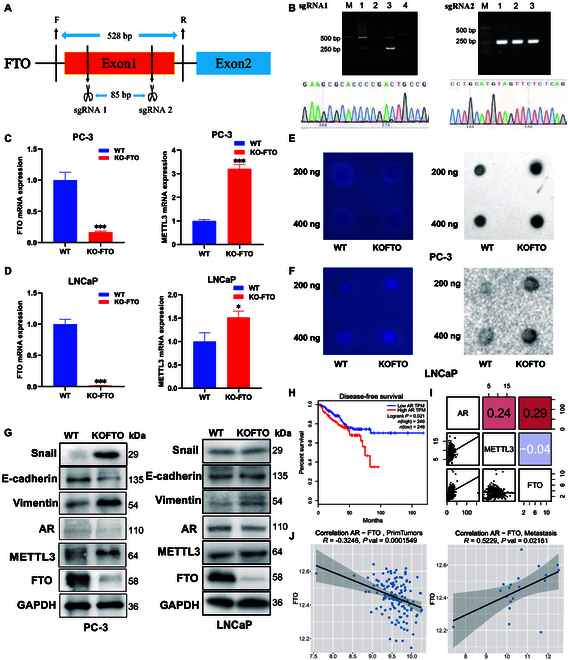
EMT in prostate cancer cells is regulated by FTO levels. (A) Schematic diagram of the CRISPR/Cas9-mediated deletion of FTO. (B) Identification of FTO deletion clones via sequencing. (C and D) FTO expression level was significantly decreased and MELLT3 expression level was significantly increased in FTO knockout cells, as demonstrated using qRT-PCR and (E and F) m6A dot blot analysis. (G) Protein level of EMT markers and m6A regulators was measured using western blot analysis. (H) Kaplan–Meier survival curves of DFS based on AR. (I) Heatmap of AR correlation with FTO and METTL3. (J) Correlation between AR and FTO expression in primary prostate cancer and metastatic prostate cancer.

The CRISPR/Cas9 gene editing system was used to generate FTO knockout cells for verifying whether the m6A levels are regulated by FTO during EMT. First, an 85-bp fragment was deleted from the first exon of FTO (Fig. 3A), and the pX458 vector was digested with BbsI and BsaI enzymes, respectively. Subsequently, monoclonal screening and colony PCR validation were performed, followed by sequencing, to verify any mismatches (Fig. 3B). The findings revealed that FTO knockout cells exhibited significantly lower levels of FTO and higher levels of METTL3 than wild-type cells (Fig. 3C, D, and G). The results also showed that FTO knockout cells exhibited considerably higher m6A levels than wild-type cells (Fig. 3E and F), thereby supporting the role of FTO as an m6A mRNA “eraser”. The protein level of both Snail and vimentin was up-regulated, whereas that of CDH1 was down-regulated in FTO knockout PC-3 cells. However, the protein level of these above EMT markers showed no difference in LNCaP cells (Fig. 3G). Next, the cell proliferation capacity of FTO knockout prostate cancer cells was evaluated in vitro. The results showed that the cell proliferation capacity was not changed in FTO knockout LNCaP cells (Fig. S2A). However, the cell proliferation capacity was enhanced in FTO knockout PC-3 cells (Fig. S2B). These findings suggest that the knockout of FTO enhances the EMT of PC-3 cells. Kaplan–Meier survival analysis based on TCGA survival data revealed that patients with prostate cancer who expressed more AR had shorter DFS (Fig. 3H). Moreover, AR expression was related to FTO expression but not to METTL3 expression (Fig. 3I). It is worth noting that there was a substantial favorable association between the levels of FTO and AR in metastatic tissues, but the observation was reversed in primary tissues (Fig. 3J). Thus, it was hypothesized that the reduced level of AR expression after the knockout of FTO in LNCaP cells inhibits the occurrence of EMT.

**Fig. 4.**
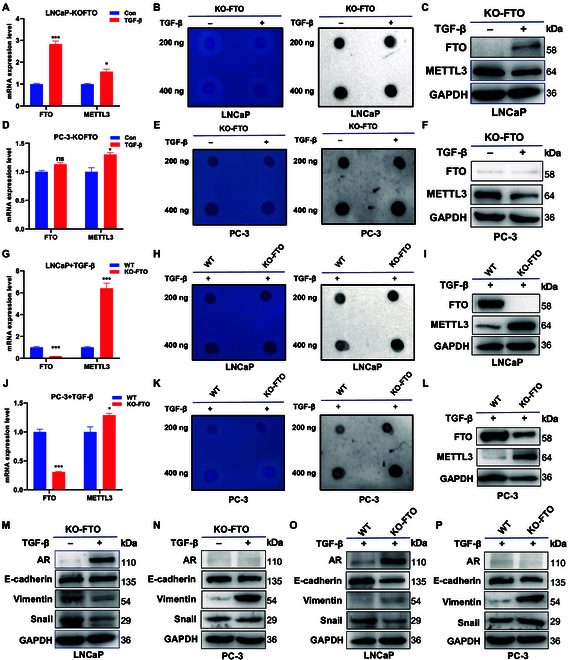
m6A level is regulated by FTO expression in EMT cells. (A) mRNA, (B) m6A, and (C) protein levels were detected in FTO knockout LNCaP cells treated with or without 10 ng/ml TGF-β for 48 h. (D) mRNA, (E) m6A, and (F) protein levels were detected in FTO knockout PC-3 cells treated with or without 10 ng/ml TGF-β for 48 h. (G) mRNA, (H) m6A, and (I) protein levels were detected in wild-type and FTO knockout LNCaP cells both treated with 10 ng/ml TGF-β for 48 h. (J) mRNA, (K) m6A, and (L) protein levels were detected in wild-type and FTO knockout PC-3 cells both treated with 10 ng/ml TGF-β for 48 h. (M) Protein level of EMT markers was detected in FTO knockout LNCaP cells and (N) PC-3 cells treated with or without 10 ng/ml TGF-β for 48 h. (O) Protein level of EMT markers was detected in wild-type and FTO knockout LNCaP cells and (P) PC-3 cells both treated with 10 ng/ml TGF-β for 48 h.

To verify the relationship among EMT, m6A level, and enzymes, FTO knockout cells were treated with/without TGF-β for 48 h. The results showed that FTO and METTL3 RNA levels were increased in TGF-β-induced FTO knockout PC-3 and LNCaP cells (Fig. 4A and D). Furthermore, TGF-β-induced reductions in m6A levels were rescued by FTO knockout (Fig. 4B and E). Western blot results showed that TGF-β treatment increased FTO and decreased METTL3 levels in FTO knockout LNCaP cells (Fig. 4C), whereas it decreased METTL3 and FTO levels in FTO knockout PC-3 cells (Fig. 4F). Prostate cancer cells with FTO knockout were pretreated with CHX and MG-132 for 6 h and then treated with or without 10 ng/ml TGF-β for 48 h, followed by the detection of METTL3 and vimentin expression using western blot analysis. The results showed that in the presence of MG-132, but not CHX, TGF-β treatment induced METTL3 expression in FTO knockout cells (Fig. S2C and D). TGF-β treatment in both wild-type and FTO knockout cells revealed that FTO knockout decreased the mRNA and protein levels of FTO and increased those of METTL3 compared with wild-type cells (Fig. 4G, I, J, and L). The m6A level was increased in FTO knockout cells compared with wild-type cells both treated with TGF-β (Fig. 4H and K). The protein level of both Snail and vimentin was down-regulated after treatment with TGF-β in FTO knockout LNCaP cells (Fig. 4M), while that of vimentin was up-regulated in FTO knockout PC-3 cells (Fig. 4N). The data also showed that in the presence of MG-132, TGF-β increased vimentin expression in FTO knockout PC-3 cells (Fig. S2E). Moreover, the protein level of vimentin was up-regulated after TGF-β treatment (Fig. 4O and P). These results indicated that the biomarkers of EMT were changed significantly under the interaction between FTO knockout and TGF-β treatment. However, the results confirmed that FTO knockout inhibited TGF-β-induced EMT in cells.

### Identification of m6A-regulated genes undergoing EMT

**Fig. 5.**
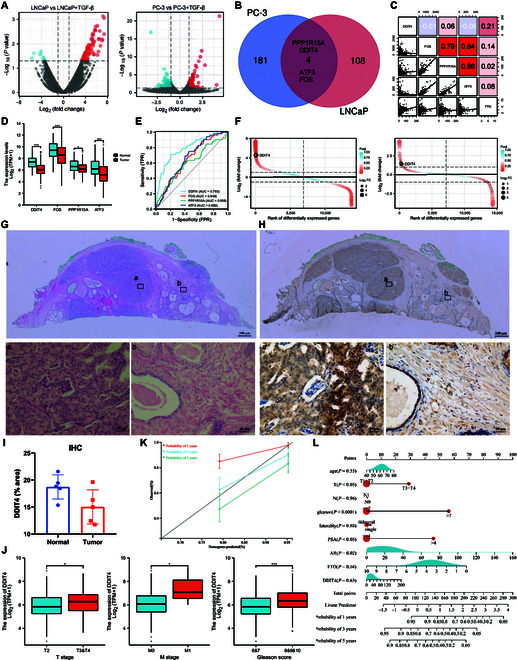
Identification of EMT-regulated genes in prostate cancer cells. (A) RNA-seq identified DEGs in EMT cells compared with wild-type cells. (B) Venn diagram summarized the common DEGs in the 2 prostate cancer cell lines. (C) Correlation of RNA expression levels between 4 DEGs and FTO. (D) RNA expression level of 4 DEGs in tumor-adjacent tissues and cancer tissues. (E) ROC curve of 4 DEGs to distinguish normal and prostate cancer tissues. (F) Ranking map of DEGs. (G) H&E staining results of prostate cancer and tumor-adjacent tissues. Representative images of tumor tissues are shown in (a) and those of tumor-adjacent tissues are shown in (b). (H) IHC (DDIT4)-stained paraffin-embedded sections obtained from patients with prostate cancer. Representative images of tumor tissues are shown in (a) and those of tumor adjacent tissues are shown in (b). (I) Quantitative IHC analysis of DDIT4. (J) Expression level of DDIT4 in different clinical groups. (K) One-, 3 -, and 5-year PFS nomograms for DDIT4. (L) The Cox method was used to construct a nomogram for prognostic evaluation.

To identify DEGs involved during EMT in prostate cancer, PC-3 and LNCaP cells were treated with TGF-β for RNA sequencing (RNA-seq). In LNCaP cells, 12 down-regulated and 100 up-regulated DEGs were identified using RNA-seq. In PC-3 cells, 97 down-regulated and 88 up-regulated DEGs were detected (Fig. 5A). Four coexpressed DEGs (ATF3, DDIT4, FOS, and PPP1R15A) were identified through the intersection of a Venn diagram (Fig. 5B). The results indicated that DDIT4 mRNA level had the highest correlation with FTO (Fig. 5C) and that the expression level of the abovementioned 4 DEGs was decreased in prostate cancer tissues. (Fig. 5D). The ROC curve showed that the area under the curve (AUC) of DDIT4 was 0.793, significantly greater than that of the other 3 genes, which helped distinguish between normal and cancer tissues (Fig. 5E). DEGs were then sorted according to |log2FoldChange|, followed by the marking of the position of DDIT4. The results demonstrated that DDIT4 expression was up-regulated in the 2 sequencing results (Fig. 5F). Normal prostate and prostate cancer tissues were differentiated using H&E staining (Fig. 5G). IHC findings revealed that DDIT4 expression was decreased in prostate cancer, whereas AR expression was up-regulated (Fig. 5H and Fig. S3A). However, DDIT4 expression was increased in the metastatic samples (Fig. S3B). Clinicopathological factor and survival analyses based on TCGA data revealed that the larger the tumor size, the higher is the Gleason score, and that patients with metastasis exhibited high DDIT4 expression (Fig. 5I). The package “rms” in R language was then used to incorporate data regarding survival duration, survival status, and 9 qualities in a column line graph for prognostic assessment using the Cox method. As per the multivariate Cox analysis of TCGA data, the tumor size (T), prostate-specific antigen, Gleason score, FTO, and AR expression levels were all associated with PFS (Fig. 5J and K). However, DDIT4 expression level was not a direct predictor of PFS. Therefore, it was necessary to clarify whether DDIT4 affects prostate cancer development via the m6A pathway.

First, DDIT4 expression level was strongly correlated to the “readers” IGF2BP2 and IGF2BP3 and the “eraser” FTO (Fig. S3C). Subsequently, DDIT4 was closely associated with the expression of m1A, m5C, and m6A regulators pan-cancer, and DDIT4 was a target gene involved in multiple modifications in prostate cancer (Fig. S4A). Using the sequence-based N6-methyladenosine modification site predictor (SRAMP) database, the m6A sites of DDIT4 were predicted to confirm the function of m6A alteration in FTO-mediated gene regulation. The “RRACH” pattern considerably increased the DDIT4 score, with the 3′ untranslated region (3′UTR) sections next to the stop codons of DDIT4 mRNA exhibiting the highest confidence in the presence of m6A alteration (Fig. S4B and C). Together, the aforementioned findings showed that FTO could regulate DDIT4 via m6A regulation.

### DDIT4 is involved in m6A-regulated EMT in prostate cancer cells

**Fig. 6.**
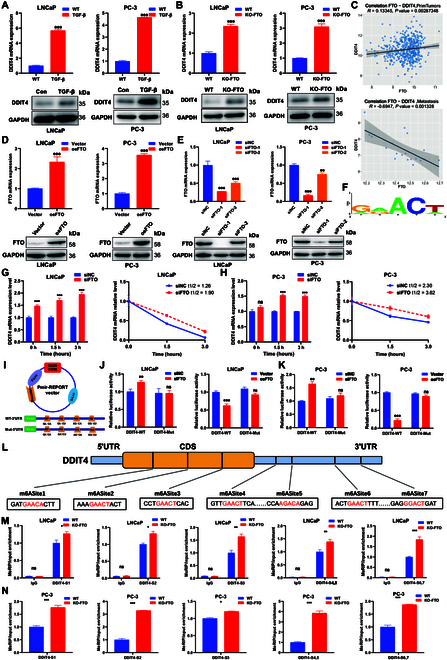
DDIT4 is involved in m6A-regulated EMT in prostate cancer cells. (A) mRNA and protein levels of DDIT4 were up-regulated in both EMT and (B) FTO knockout cells. (C) Correlation between DDIT4 and FTO expression in primary prostate cancer and metastatic prostate cancer. (D) FTO overexpression in LNCaP and PC-3 cells. (E) FTO silencing reduced FTO expression level. (F) Predominant consensus motif was detected using the SRAMP database. (G) mRNA half-lives were estimated after the indicated actinomycin D treatment in FTO silencing LNCaP and (H) PC-3 cells. (I) Dual fluorescent vector ligation and site-specific mutation map. (J and K) Relative luciferase level of wild-type DDIT4, but not of the mutated gene, was significantly increased in FTO silencing LNCaP and PC-3 cells; the opposite results were observed in the FTO overexpression group. (L) Schematic representation of the location of DDIT4 m6A motif sites. (M) FTO knockout in LNCaP cells increased the level of DDIT4 m6A modification. (N) FTO knockout in PC-3 cells increased the level of DDIT4 m6A modification.

The DDIT4 level was increased in both EMT and FTO knockout cells, consistent with the RNA-seq results (Fig. 6A and B). Of note, there was a positive association between the expression level of FTO and DDIT4 in primary tissues; the association was reversed in metastatic tissues (Fig. 6C). The mRNA and protein levels of FTO were up-regulated after FTO overexpression but down-regulated after its silencing, and its results verified using qRT-PCR and western blot (Fig. 6D and E). The RNA and protein levels of FTO were down-regulated after silencing FTO; the effect of siFTO-1 was significantly better than that of siFTO-2. Therefore, siFTO-1 was selected for subsequent experiments. The GGAC/AGAC/GAAC motif highly enriched within m6A locations was predicted using the SRAMP database (Fig. 6F). In addition, the rate of RNA degradation in silent and knockout FTO prostate cancer cells and corresponding controls was measured. The results revealed that DDIT4 mRNA level was increased and that the mRNA half-life was prolonged after FTO silence and knockout in LNCaP and PC-3 cells (Fig. 6G and H and Fig. S4D and E). Luciferase reporter experiments were performed using constructs containing either wild-type or mutant DDIT4 m6A sites in the 3′UTR to examine the effects of m6A modification on DDIT4 expression and evaluate the impact of target m6A mRNA alteration on gene regulation. m6A alteration was eliminated from the altered DDIT4 constructs by replacing adenosine bases (A) in the m6A consensus sequences (RRACH) with thymine bases (T). Luciferase reporter experiments showed that the m6A modification of DDIT4 in the wild-type, but not in the mutated type, was significantly increased in cells treated with siFTO and FTO knockout (Fig. 6I and Fig. S4F and G); opposite results were observed in the FTO overexpression group (Fig. 6K). MeRIP-qPCR was used to assess the m6A level of DDIT4 in FTO knockout LNCaP and PC-3 cells. Previously, DDIT4 was shown to have 9 m6A sites using the SRAMP website. Of the 9, 7 had very high scores: 3 were in the coding sequence (CDS) region and 4 were in the 3′UTR region. Therefore, MeRIP-qPCR was used to verify these 7 m6A motif sites. The findings demonstrated that m6A abundance was significantly elevated in DDIT4 mRNA after FTO knockout in LNCaP and PC-3 cells (Fig. 6L to N).

### m6A “readers” IGF2BP2 and IGF2BP3 regulate DDIT4 function

**Fig. 7.**
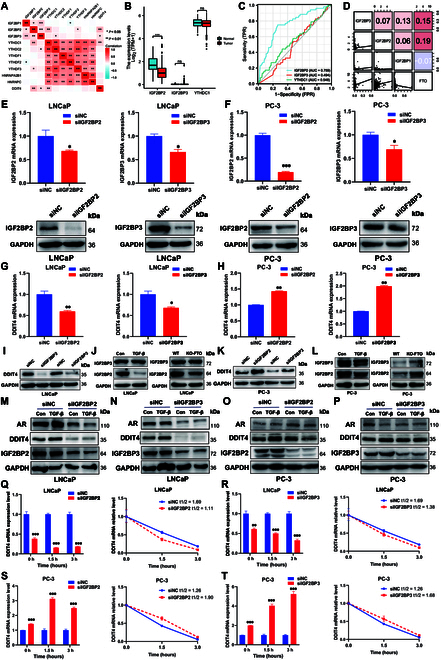
Screening for “readers” involved in regulating the target gene DDIT4. (A) Heat map of the correlation between the expression level of m6A “readers” and DDIT4. (B) RNA expression level of 3 “readers” in tumor-adjacent tissues and cancer tissues. (C) ROC curve of 3 “readers” to distinguish normal and prostate cancer tissues. (D) Correlation of RNA expression level between IGF2BPs and DDIT4. (E) mRNA and protein levels of IGF2BP2 and IGF2BP3 were decreased after IGF2BP2 and IGF2BP3 silencing in LNCaP and (F) PC-3 cells. (G) RNA expression level of DDIT4 was decreased after IGF2BP2/3 silencing in LNCaP cells. (H) RNA expression level of DDIT4 was increased after IGF2BP2/3 silencing in PC-3 cells. (I) Protein expression level of DDIT4 was decreased after IGF2BP2/3 silencing in LNCaP cells. (J) Protein expression level of IGF2BP2/3 after treatment with TGF-β or FTO knockout in LNCaP cells. (K) Protein expression level of DDIT4 was increased after IGF2BP2/3 silencing in PC-3 cells. (L) Protein expression level of IGF2BP2/3 after treatment with TGF-β or FTO knockout in PC-3 cells. (M) Protein expression was measured using western blot analysis after pretransfection with siNC or si-IGF2BP2 (N)/si-IGF2BP3 for 12 h. LNCaP and (O and P) PC-3 cells were further treated with or without 10 ng/ml TGF-β for 48 h. (Q) mRNA half-lives were estimated after the indicated actinomycin D treatment in siIGF2BP2/ (R) siIGF2BP3 LNCaP and (S and T) PC-3 cells.

In a previous study, the m6A “eraser” FTO was shown to regulate the m6A modification level of the EMT target gene DDIT4 in prostate cancer. However, it was unclear which “readers” recognized DDIT4 function. Thus, it was first found that DDIT4 expression level was closely related to the recognition proteins IGF2BP2, IGF2BP3, and YTHDC1 (Fig. 7A). Among these 3 genes, only IGF2BP2 expression was down-regulated in prostate cancer (Fig. 7B), and the ROC curve showed that the AUC of IGF2BP2 was higher than that of the other 2 genes for distinguishing between normal and cancer tissues (Fig. 7C). The IGF2BP family, which acts as m6A “readers”, detects m6A-methylated mRNA and controls target mRNA stability.

The results showed that the mRNA level of DDIT4 was associated with IGF2BP2 and IGF2BP3 (Fig. 7D). The expression of IGF2BP2/3 in LNCaP and PC-3 cells was silenced using 2 IGF2BP2/3-targeting small interfering RNAs (siRNAs) to confirm their involvement in m6A-regulated DDIT4 expression. The reduced effectiveness was then validated using qRT-PCR and western blot (Fig. 7E and F). IGF2BP2/3 silencing significantly reduced the mRNA and protein expression of DDIT4 in LNCaP cells but increased the expression in PC-3 cells (Fig. 7G to I and K). Subsequently, the protein expression level of IGF2BP2 was found to be reduced after FTO knockout in LNCaP cells, whereas the expression level of IGF2BP2 was increased in PC-3 cells (Fig. 7J and L). After pretransfecting LNCaP and PC-3 cells with siNC or si-IGF2BP2/3 for 12 h, the cells were treated with or without TGF-β. Western blot analysis used to determine protein expression demonstrated that si-IGF2BP2/3 inhibited the TGF-β-induced production of DDIT4 in prostate cancer cells (Fig. 7M to P). Moreover, the measurement of the rate of RNA decay in prostate cancer cells with IGF2BP2/3 silencing and corresponding controls showed that the DDIT4 mRNA level was decreased and the mRNA half-life was significantly shortened after IGF2BP2/3 silencing in LNCaP cells (Fig. 7Q and R). However, DDIT4 stability observed after siIGF2BP2/3 treatment in PC-3 cells was reversed in LNCaP cells (Fig. 7S and T). Together, these results revealed that m6A “readers” are involved in regulating the expression and EMT function of DDIT4.

### DDIT4 participates in cell proliferation and migration

**Fig. 8.**
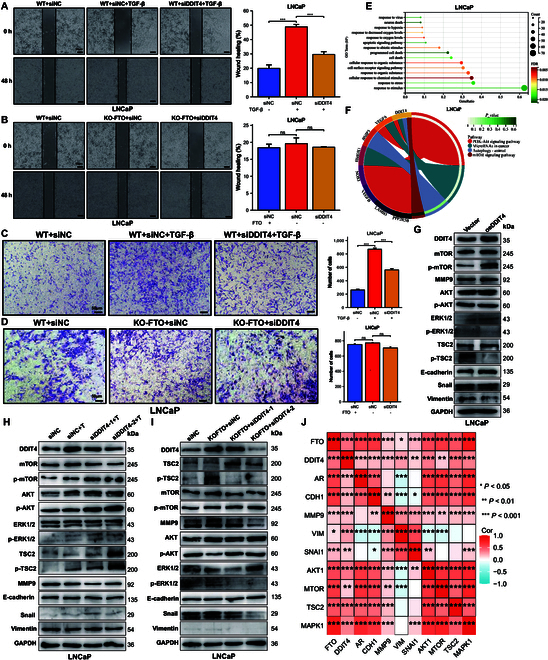
Effect of DDIT4 expression level on cell biological characteristics in LNCaP cells. (A) Wound-healing assays were performed to determine the effect of TGF-β and siDDIT4 and (B) that of FTO knockout and siDDIT4 on LNCaP cells. (C) Transwell assays were performed to determine the effect of TGF-β and siDDIT4 and (D) that of FTO knockout and siDDIT4 on LNCaP cells. (E) Top 15 BP terms, including DDIT4, enriched in LNCaP cells. (F) KEGG pathway genes, including DDIT4, enriched in LNCaP cells. (G) Protein expression of genes in the PI3K-AKT-mTOR pathway was measured using western blot analysis after DDIT4 overexpression. (H) Protein expression of genes in the PI3K-AKT-mTOR pathway was measured using western blot analysis after siDDIT4 and TGF-β treatment. (I) Protein expression of genes in the PI3K-AKT-mTOR pathway was measured using western blot analysis after siDDIT4 in FTO knockout cells. (J) Correlation analysis among FTO, DDIT4, and AR, and the expression levels of pathway genes and EMT genes.

An in-depth investigation was conducted into the functions of DDIT4 in the FTO-triggered EMT of prostate cancer cells, even though the promotion roles of FTO/TGF-β in EMT and prostate cancer development have been extensively established previously. The down-regulated expression of DDIT4 reduced the promotion of wound healing after TGF-β treatment in both cell lines (Fig. 8A and Fig. S5A), and the same effect was observed after FTO knockout in PC-3 cells but not following FTO knockout in LNCaP cells (Fig. 8B and Fig. S5B). The results of transwell assays were consistent with those of wound-healing assays (Fig. 8C and D and Fig. S5C to D). Therefore, DDIT4 may be involved in the m6A-regulated EMT invasion and migration of prostate cancer.

The RNA-seq results were next analyzed using Gene Ontology (Fig. 8E and Fig. S5E) and KEGG enrichment (Fig. 8F and Fig. S5F) analyses. KEGG enrichment analysis showed that DDIT4 in the RNA-seq results of the 2 cell lines was enriched in 4 pathways, including microRNA in cancer, phosphoinositide 3-kinase (PI3K)-Akt signaling pathway, mammalian target of rapamycin (mTOR) signaling pathway, and autophagy-animal. However, only the PI3K-Akt signaling pathway was statistically significant. Therefore, western blotting was used to determine how DDIT4 affects the PI3K-Akt-mTOR signaling pathway during prostate cancer EMT. First, DDIT4 overexpression in cells did not significantly transform TSC2 levels, but TSC2 phosphorylation was significantly decreased in both cell lines. However, the change in TSC2 phosphorylation level possibly blocked the PI3K-AKT-mTOR pathway (Fig. 8G and Fig. S6A). Second, the 2 aforementioned siRNAs were transformed in the cells, which revealed that the effect of siDDIT4-2 was better than that of siDDIT4-1. The results also showed that DDIT4 expression was increased after the addition of TGF-β in the 2 cell lines and that DDIT4 expression was decreased after DDIT4 silencing. Finally, there was no discernible difference in the amount of mTOR expression after siDDIT4 treatment, whereas mTOR phosphorylation was significantly increased in both cell lines (Fig. 8H and Fig. S6B).

DDIT4 expression level was significantly increased after FTO knockout. Therefore, the protein level of the pathway- and EMT-associated genes after DDIT4 silencing was measured in FTO knockout cells. TSC2 expression and phosphorylation level were decreased in FTO knockout PC-3 cells, whereas siDDIT4 restored the expression and phosphorylation level. However, TSC2 phosphorylation was up-regulated after FTO knockout in LNCaP cells; siDDIT4 could restore the phosphorylation level (Fig. 8I and Fig. S6C). DDIT4 expression in prostate cancer was closely associated with PI3K-AKT-mTOR pathways node gene and EMT biomarker expression (Fig. 8J). Previous findings have revealed that DDIT4 expression can lead to the negative regulation of the PI3K-AKT-mTOR signaling pathway, whereas its overexpression can exhibit protective and damaging effects on cells under oxidative stress [20]. In this section, change in DDIT4 expression was found to reverse TSC2 expression level after FTO knockout cells underwent EMT. Moreover, it had a rescue effect on vimentin expression. However, the role of oxidative stress on cells was not evaluated in this study. Thus, DDIT4 may play a more important role in hypoxia, but this hypothesis needs additional verification.

**Fig. 9.**
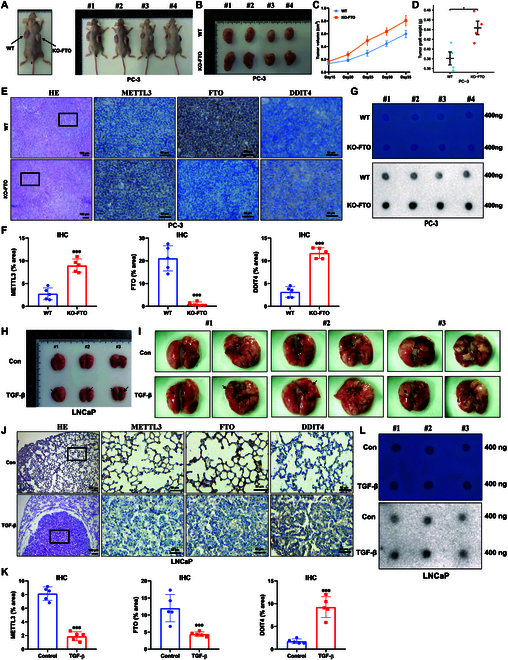
TGF-β treatment and FTO knockout enhances tumor growth and metastasis in vivo. (A) FTO knockout and wild-type PC-3 cells were injected into the flanks of nude mice; the representative mice are presented. (B) The treated BALB/c nude mice were sacrificed for their xenografts, and the tumor size was measured using a ruler. (C) Tumor growth curve of xenografts was plotted in the wild-type and FTO knockout groups using a Vernier caliper. (D) Tumor weight was measured in wild-type and FTO knockout groups. (E) H&E and IHC staining micrographs of the protein levels in tumor xenografts. (F) Quantitative IHC analysis of METTL3, FTO, and DDIT4 after FTO knockout. (G) m6A level was measured in wild-type and FTO knockout groups. (H and I) LNCaP wild-type cells and LNCaP cells treated with TGF-β were injected into nude mice via tail vein injection. Representative images of metastatic lung tumors. (J) H&E and IHC staining micrographs of the protein levels in lung tissues. (K) Quantitative IHC analysis of METTL3, FTO, and DDIT4 after treatment with TGF-β. (L) m6A level was measured in the wild-type and EMT groups.

Wild-type and FTO knockout PC-3 cells were used to establish an animal xenograft model, and the results showed that FTO knockout PC-3 cells exhibited a substantial increase in tumor development over 35 d of flank xenograft measurement in BALB/c nude mice (Fig. 9A). The xenografts in nude mice were subsequently dissected and isolated (Fig. 9B). The tumor volume was significantly larger in the FTO knockout group than in the control group (Fig. 9C), in line with the growth rate tested via tumor weights (Fig. 9D). In addition, the total m6A level was assessed in the retrieved xenografts, and the findings showed that FTO knockout significantly increased the total m6A level in vivo (Fig. 9E). H&E and IHC labeling was employed to validate the expression of FTO, METTL3, and the FTO-targeted gene DDIT4 in xenograft tissues, and the results showed that the FTO protein level was suppressed and METTL3 and DDIT4 expression levels were up-regulated (Fig. 9F).

LNCaP is a low-metastatic prostate cancer epithelial cell line. According to literature, LNCaP cells have a low degree of malignancy, have no subcutaneous tumor, and do not easily lead to lung metastasis. Therefore, LNCaP cells were selected and treated with TGF-β to construct an EMT model of lung metastasis. As shown in Fig. 9G and H, the number of lung tumors produced by TGF-β-treated LNCaP cells was significantly higher than that by wild-type cells, indicating that EMT encouraged tumor metastasis in vivo. In addition, the total m6A level in the group treated with TGF-β was up-regulated (Fig. 9I). Because there was almost no tumorigenic tissue in the wild-type group, the control group contained almost all normal lung tissue. H&E used to verify the results of tumorigenesis in vivo (Fig. 9J) revealed that the wild-type group was normal lung tissue, whereas the TGF-β group contained tumor tissue. IHC performed to assess changes in gene expression levels found that METTL3 and FTO expression in the TGF-β group was decreased and DDIT4 expression was increased (Fig. 9J). The above H&E and IHC results were evaluated by 2 professional pathologists.

### FTO and DDIT4 are involved in prostate cancer bone metastasis

**Fig. 10.**
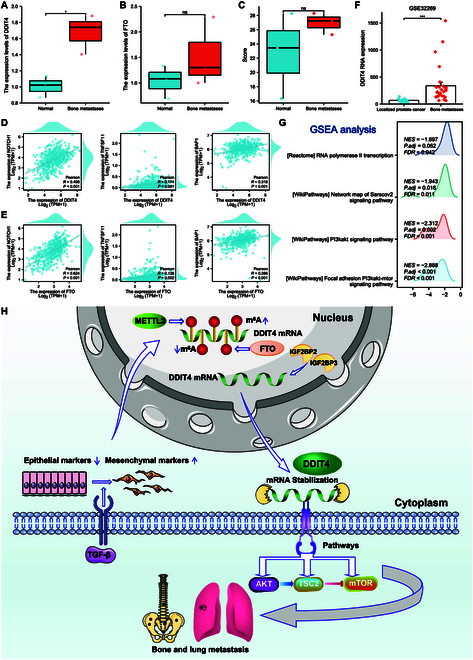
FTO and DDIT4 are involved in prostate cancer bone metastasis. (A) RNA expression level of DDIT4 and (B) FTO in the bone metastasis and control groups. (C) TPSA level in the bone metastasis and control groups. (D) Correlation analysis between the expression level of DDIT4 and bone metastasis markers. (E) Correlation analysis between the expression level of FTO and bone metastasis markers. (F) RNA expression level of DDIT4 in the GSE32269 dataset. (G) Pathway enrichment of DDIT4 in the GSE32269 dataset. (H) All findings in this study are depicted as a schematic diagram.

The present study provided further evidence that variations in FTO and DDIT4 expression level correlated with prostate cancer tumor development and metastasis both in vitro and in vivo. An association was detected between m6A methylation and bone metastasis in prostate cancer, which showed that FTO knockout boosted DDIT4 expression and stimulated PC-3 cell invasion and EMT in vivo. First, bone ECT was combined with CT to determine bone metastasis in prostate cancer. The red arrow in the left image (Fig. S7A, C, and E) of Fig. S6 indicates bone metastasis, whereas the right image (Fig. S7B, D, and F) is the control group without metastasis. Three prostate cancer bone metastasis tissues and 3 primary prostate cancer tissues were selected for FTO, DDIT4, and total PSA detection. The findings demonstrated that DDIT4 expression in the bone metastasis group was up-regulated, consistent with the results of in vitro experiments (Fig. 10A to C). Recent research has revealed that the expression level of NOTCH1, BAP1, and TNFSF11 is closely related to bone metastasis in prostate cancer. Therefore, the relationship among DDIT4, FTO levels, and the bone metastasis markers NOTCH1, BAP1, and TNFSF11 was assessed using the TCGA database. The findings demonstrated a favorable correlation of the expression level of NOTCH1/BAP1/TNFSF11 with FTO/DDIT4 (Fig. 10D and E). However, owing to the limited clinical samples included in this study, the GSE32269 dataset from the Gene Expression Omnibus database, containing 22 prostate cancer tissues and 29 bone metastases tissues, and the same results to our data (Fig. 10F). RNA-seq revealed that DDIT4 expression was markedly up-regulated in the bone metastasis group and that DDIT4 was enriched in the PI3K-AKT-mTOR signaling pathway (Fig. 10G). These RNA-seq results at the tissue level were consistent with the RNA-seq results obtained at the prostate cancer cell line level.

Taken together, m6A regulated the occurrence and metastasis of prostate cancer and its levels in the 2 processes exhibited a completely opposite trend. FTO, as a demethyltransferase, inhibited the m6A level in prostate cancer and induced EMT, thereby playing a dual role. The m6A motif modification of DDIT4 was regulated by FTO expression and the half-life and function of DDIT4 were regulated by the “readers” IGF2BP2 and IGF2BP3. Finally, the DDIT4 expression level was associated with bone metastasis in prostate cancer. A schematic diagram has been drawn to represent all of the study findings (Fig. 10H).

## Discussion

Prostate cancer, which has a low metastasis rate, is a common cancer in men. However, some patients with a highly aggressive disease rapidly develop metastatic tumors, which are resistant to treatment and cause cancer-specific death. Current studies have shown that EMT is a crucial mechanism that initiates the metastatic process. Among the various factors involved, TGF-β is one of the key players in the process of inducing EMT. TGF-β is a tumor suppressor that promotes apoptosis and differentiation in both healthy and precancerous cells [21]. However, cells lose their inhibitory properties and adopt a proliferative phenotype during tumor development, thereby initiating immune escape and growth factor expression, ultimately leading to EMT [22]. In this study, an EMT model was induced in prostate cancer cells by exploiting this property of TGF-β. The m6A modification level was significantly reduced in EMT cells compared with wild-type cells, which was caused by increased FTO expression. FTO knockout, however, inhibited the effect of TGF-β on the decrease in the m6A modification level. EMT only occurred in PC-3 cells after FTO knockout, whereas the migration and invasion abilities LNCaP cells were not significantly changed. Thus, differences in the biological function between the 2 cells after FTO knockout may be associated with AR expression level.

Studies [23] have found that almost all m6A regulators are associated with AR expression. At present, m6A regulators can be used as early diagnostic markers to supplement PSA diagnosis, which can help improve the diagnosis rate of prostate cancer. Under the conditions of AR pathway inhibition, m6A-modified AR mRNA was shown to convert from actively translating polysomes to RNA protein stress granules, resulting in the reduced translation of AR mRNA. Previous research has revealed that the interaction between the TGF-β and androgen axis could regulate AR expression level, leading to AR-dependent outcomes, which may be a key factor that affects EMT [24]. Meanwhile, the androgen signaling pathway can increase Snail expression level [25,26], which in turn can inhibit E-cadherin expression in various cancer cell types [27,28]. After treatment with either 5-dihydrotestosterone alone or in combination with TGF-β, Snail expression was significantly increased in TGF-β-sensitive LNCaP-TRII cells, with the results indicating that AR independently promoted EMT by avoiding TGF-β-induced effects [6]. In the current study, FTO knockout reduced the AR expression level in LNCaP cells and led to no discernible transition in Snail and E-cadherin expression. Thus, it was speculated that the inhibition of AR expression suppresses the occurrence of EMT in LNCaP cells. In addition, the present study results confirmed that FTO down-regulation was linked to poor prostate cancer prognosis and FTO knockout in AR-negative PC-3 cells promoted cell proliferation and migration via EMT. Moreover, FTO expression significantly increased following EMT, and FTO knockout rescued Snail and E-cadherin expression and suppressed the EMT process. Jeschke et al. [15] found that FTO was down-regulated in breast cancer and that it increased the methylation level of m6A in the Wnt pathway, which triggered EMT. The current study results are consistent with their results.

In recent years, increasing evidence has shown that EMT is not a discrete process but that it reactivates some indicators of cancer through a series of intermediate states [29]. In acute myeloid leukemia, FTO was shown to control stem cell differentiation through the ASB2/RARA axis, and FTO was revealed to affect immunotherapy by regulating intracellular factors (PD-1, CXCR4, and SOX10) in melanoma. These studies [30,31] suggested that FTO regulated m6A levels involved in numerous multiple cellular procedures and that differences in these processes explained the complex dual regulation of FTO in cancer. Finally, the balance between FTO acting as a tumor promoter and suppressor may be influenced by other factors, such as mutations and the altered expression levels of key transcription factors, which are very reliant on the tissue type or even the different cancer types. Indeed, this dependence has been demonstrated in epithelial cell tumors. The current research suggested that FTO not only has a tumor suppressing function in prostate cancer but also a dual function as an m6A “eraser” in this disease. However, this complexity is increased by the diversity of m6A functions and mRNA methylation. Using RNA-seq, the EMT target gene DDIT4 was identified to be regulated by the m6A level. The suppression of METTL3 expression delayed mRNA export, whereas the inhibition of ALKBH5 expression accelerated mRNA export, which is known to be facilitated by m6A. In addition, FTO has been shown to inhibit ASB2 and RARA stability [32]. Next, the interference of FTO expression in prostate cancer cells showed that the DDIT4 mRNA half-life was longer in cancer cells than in the wild-type cells, which indicated that m6A had a positive regulatory effect on mRNA stability. Dual-fluorescence assay and MeRIP-qPCR revealed that the m6A motif in the CDS region and 3′UTR of DDIT4 was regulated by FTO expression level. These results suggest that DDIT4 is an EMT target gene regulated by m6A.

m6A switch-regulated splicing regulators and the m6A “reader” HNRNPC may have an impact on the alternative splicing of target mRNAs [33]. Moreover, the deacetylation of RNA promoted by the m6A “reader” YTHDF leads to the degradation of mRNA. DDIT4 expression was most closely related to the IGF2BP family among the numerous “readers”. Under normal conditions, IGF2BPs promote mRNA stability by binding to mRNA stabilizers. However, under stress conditions, IGF2BPs enhance RNA expression by facilitating their storage via their trafficking to stress granules. IGF2BP2 recognizes m6A in the CDS region of SOX2 and prevents its degradation, thereby contributing to the development and incidence of colorectal cancer. IGF2BP3 has carcinogenic characteristics and is significantly up-regulated in numerous cancer subtypes, associating with poor survival. In this study, the inhibition of IGF2BP2/3 expression revealed that the half-life of DDIT4 mRNA in PC-3 cells after IGF2BP2/3 silencing was longer than that in wild-type cells, which indicated that IGF2BP2/3 negatively regulated the stability of mRNA in PC-3 cells. By contrast, IGF2BP2/3 had a positive regulatory effect on mRNA stability in LNCaP cells. The RNA expression trend of IGF2BP2/3 in different cells was consistent with that of AR. It has been speculated that the role of IGF2BP2/3 in regulating the stability of target genes in prostate cancer may be affected by the expression level of AR, but the precise mechanism remains unclear.

The most well-known function of DDIT4 is inhibiting the proliferation signal pathway and regulating metabolism by inhibiting the regulatory factor mTOR in the pathway. Cancer frequently hyperactivates the PI3K/AKT/mTOR signaling pathway, which controls several cellular activities, including cell growth [34], proliferation [35], motility, survival, and apoptosis [36]. In addition, DDIT4 is a key effector of autophagy, thereby promoting prostate cancer resistance to proteasome inhibitors (bortezomib) by regulating the formation of autolysosomes. In the current study, DDIT4 was primarily enriched in the PI3K/AKT/mTOR signaling pathway associated with cell proliferation. The TSC1/2 complex, located at the junction of the mTORC1 signaling pathway, acts as a key integron that controls the positive and negative signals of cell metabolism. Thus, DDIT4 can directly act on the TSC1/2 complex in the PI3K/AKT/mTOR pathway. Some studies have shown that DDIT4 acts as an effective cell growth inhibitor in a tuberous sclerosis 1/2 (TSC1/2)-dependent manner, induced by the overexpression of the downstream activated kinases of PI3K: protein kinase B (PKB) and phosphoinositide-dependent protein kinase 1 (PDK1) [37]. However, the effect of DDIT4 on TSC2 or 14-3-3 protein and mTORC1 requires that DDIT4 should be able to bind 14-3-3. In addition, the effects of DDIT4 are unrelated to the modifications in AKT phosphorylation or TSC2 phosphorylation stimulated by AKT in hypoxia. Therefore, in the presence of numerous growth factors, DDIT4 disrupts the ability of TSC2 to bind to 14-3-3 and provides a mechanism for quickly extinguishing mTORC1 activity under hypoxic conditions. Taken together, the mechanism through which DDIT4 regulates the PI3K/AKT/mTOR pathway via TSC2 is influenced by many factors and specific conditions. The current study showed that DDIT4 expression led to a change in TSC2 expression; however, its direct effect on mTOR was weak. Moreover, the sequencing results showed that although DDIT4 was enriched in the PI3K/AKT/mTOR pathway, *P* values in one group were >0.05, indicating that DDIT4 may lack the ability to bind 14-3-3 during EMT or may not have effect on the PI3K/AKT/mTOR pathway because of differences in oxygen conditions.

There are several limitations in this study. The study demonstrated that the m6A level was attenuated in TGF-β-induced EMT models and that FTO knockout inhibited these effects. DDIT4, a target gene of EMT that is regulated by FTO, is involved in prostate cancer metastasis. However, because of limited clinical data, the relationship between DDIT4 expression and bone metastasis could not be adequately investigated in this study. Thus, larger sample sizes are needed for clinical investigation and the in-depth analysis of mechanisms.

In summary, m6A methylation can be detected in almost all RNA types, and it plays an important role in development, organism homeostasis, and disease. In this study, in vivo and in vitro experiments revealed that m6A levels regulated the occurrence of prostate cancer and progression of EMT. Changes in m6A levels during tumor occurrence and metastasis were shown to be dependent on the regulation of the expression level of demethyltransferase FTO. Moreover, RNA-seq and MeRIP-qPCR showed that DDIT4 was a key target gene involved in m6A level-regulated EMT. It should be emphasized that various prostate cancer cells may react differently to DDIT4 mRNA that has been modified by FTO/IGF2BP2/IGF2BP3 and that although DDIT4 is believed to participate in proliferation-related signaling pathways, its regulatory mechanism is complex and there are many interfering factors. Thus, the specific mechanism of DDIT4 participate in pathways needs further investigation.

## Methods

### Cell culture, treatment, and transfection

PC-3 and LNCaP cells (from American Type Culture Collection cell bank) were cultured in RPMI 1640 (Gibco, USA) supplemented with 10% fetal bovine serum (FBS) (CellMax, China) and 1% penicillin–streptomycin (Solarbio, China) at 37 °C in an incubator with 5% CO_2_ humidity. The EMT model of prostate cancer was established by treating cells with 10 ng/ml TGF-β (Peprotech, China) for 48 h. pcDNA/FTO was kindly provided by Dr. Xin Liu at the Harbin Institute of Technology and pcDNA/DDIT4 or vector control was provided by Fenghui organism (China). siRNAs against IGF2BPs, DDIT4, and FTO were prepared by Gene Pharma (Suzhou, China). siRNA/pcDNA was transfected into cells using Lipofectamine 3000 (Invitrogen). The siRNA sequences are listed in the supplementary information (Table S2).

### Patient samples and IHC

Samples from patients with prostate cancer were collected at the Xiang’an Hospital of Xiamen University. Sections were microwave-heated in sodium citrate buffer for antigen retrieval, deparaffinized in xylene, and hydrated with gradient ethanol. The sections were treated with the relevant antibodies at 4 °C overnight, followed by blocking in 2% bovine serum albumin. The sections were then treated with a rabbit/mouse secondary antibody and incubated in the dark for 60 min at room temperature. The results were observed using Vectastatin DAB kit (ZSGB-BIO, China). Two independent scientists scored the staining intensity in a blinded manner.

### RNA extraction and qRT-PCR

Total RNA, extracted using RNAiso Plus (TaKaRa, China), was reverse-transcribed into cDNA using PrimeScript RT Reagent Kit. The RNA expression level was evaluated using qRT-PCR, and the results of the target gene were computed using the 2^−ΔΔCt^ technique with GAPDH as the control. The primers for the targeted genes are listed in Table S1.

### METTL3 knockout

The CRISPR/Cas9 gene editing technique was used to generate FTO knockout PC-3 and LNCaP cells. The sgRNA sequences are listed in Table S2. The px458 vector was first used to insert the annealed double-stranded DNA in the BbsI and BsaI (NEB, USA) restriction sites. Then, Lipofectamine 3000 was used to transfect cells with the purified recombinant plasmid. The cells were seeded into 96-well plates and collected for testing the knockdown efficiency after puromycin screening. Finally, FTO knockout cells were selected for culture and subsequent experiments.

### Western blot analysis

Proteins were first extracted using the radioimmunoprecipitation assay lysis solution with phenylmethylsulfonyl fluoride and protease inhibitor cocktail (APEXBIO, USA) and then measured using a bicinchoninic acid kit (Beyotime, China). SDS-polyacrylamide gel electrophoresis was used to separate the protein lysates, which were then transferred to polyvinylidene fluoride membranes. After blocking the membranes with 5% skim milk, they were incubated with specific primary antibodies overnight at 4 °C, followed by a 1-h incubation with secondary antibodies at room temperature. On the Mini-REPORT Tetra Electrophoresis System, proteins isolated on the membranes were visualized using an ECL chromogenic kit (Beyotime, China). The antibodies used in this study were as follows: FTO (Abcam; ab126605, 1:1,000), m6A (Abcam; ab286164, 1:1,000), METTL3 (Abcam; ab69325, 1:1,000), vimentin (CST; 5741T, 1:1,000), CDH1 (CST; 3195S, 1:1,000), Snail (Wanlei; WL01863, 1:1,000), IGF2BP2 (Proteintech; 11601-1-AP, 1:1,000), IGF2BP3 (Proteintech; 14642-1-AP, 1:1,000), DDIT4 (Proteintech; 10638-1-AP, 1:1,000), AKT (CST; 4685S, 1:1,000), TSC2 (Proteintech; 24601-1-AP, 1:1,000), p-AKT (CST; 13038S, 1:1,000), p-TSC-2 (Proteintech; 29000-1-AP, 1:1,000), AR (Proteintech; 22089-1-AP, 1:1,000), and GAPDH (Proteintech; HRP-60004, 1:1,000).

### m6A dot blot analysis

The total RNA concentration was adjusted to a fixed value of 800, 600, or 400 ng/μl and then placed in a metal bath at 95 °C for 5 min, followed by incubation on ice for 10 min until the solution was completely cooled. After mixing RNA with 20× saline sodium citrate (SSC) 1:1, the samples were added to the nitrocellulose filter membrane according to the concentration gradient and then an ultraviolet glue was attached for 15 min. The bonded nitrocellulose filter membrane (NC) was placed in the aqua blue solution for 2 min and then washed with TBS-T for cleaning and subsequent imaging. The subsequent steps are similar to that for western blot analysis.

### RNA-seq and data analysis

PC-3 and LNCaP cells were treated with 10 ng/ml TGF-β for 48 h. Total RNA was isolated from wild-type and EMT cells using TRIZOL and transferred to Shanghai Personal Biotechnology Co., Ltd. (Shanghai, China) for RNA-seq. DEGs between the 2 groups were screened using the “limma” package of the R language. The |log2FC| of >1 and adjusted *P* value of <0.05 were regarded as statistically significant. R language was used for DEG enrichment analysis.

### m6A MeRIP-qPCR

In this study, an m6A MeRIP kit (GenSeq, China) was used to perform m6A immunoprecipitation-related experiments. The starting sample type of this kit uses >100 μg of total RNA or > 3 μg purified mRNA. For the study, >100 μg of total RNA was used as the starting sample. After determining RIP enrichment using qPCR, MeRIP was performed using GenSeq MeRIP m6A kit as per the manufacturer’s instructions. After normalizing to the input sample, the amount of m6A enrichment in each sample was determined. Table S3 contains the list of the qPCR primers.

### Luciferase reporter assay

Wild-type and mutant segments were synthesized using m6A motifs in DDIT4-3′UTR. Then, the segments were inserted into the pMIR-REPORT vector for the dual-luciferase reporter assays. Prostate cancer cells seeded in 24-well plates were cotransfected with DDIT4 fragments, FTO knockout/pcDNA3.1-FTO, and pRL-TK (Renilla luciferase control reporter vector). The cells were lysed with passive lysis buffer and collected 48 h after transfection. The relative luciferase activity of the sample was calculated via Firefly/Renilla, and each group experiment was performed in triplicate.

### RNA stability

Prostate cancer LNCaP and PC-3 cells were seeded in 6-well plates and transfected with siRNA for 24 h. After 48 h of transfection, 2 μg/ml actinomycin D was added to each well, which was considered 0 h. RNA was subsequently collected at 0, 1.5, and 3 h and extracted and quantitatively analyzed after reverse transcription. The half-life changes in mRNA were calculated using quantitative analysis.

### Protein stability

CHX and MG132 were added to PC-3 cells treated with/without TGF-β and FTO knockout PC-3/LNCaP cells treated with/without TGF-β at the indicated times to measure protein stability. Western blot analysis was used to evaluate the expression of METTL3, CDH1, and vimentin.

### Wound-healing, cell proliferation, and in vitro invasion assays

Cells were evenly spread in 12-well plates, and 1 ml of medium was added to each well for wound-healing assay. With the tip of a 200-μl pipette, the single-layer cells were scratched with the numeral “one”, cleaned with PBS 3 times, and observed under an inverted fluorescence microscope and photographed. The cells were then cultured in FBS-free media. After 48 h, the samples were observed under inverted fluorescence microscope again.

Cells were seeded in 96-well plates and incubated overnight for the cell proliferation experiment. Then, the cells with or without TGF-β treatment were assessed for cell viability using MTT for 0, 12, 24, 36, and 48 h. In wild-type and FTO knockout cells, cell viability was assessed for 0, 24, 48, 72, and 96 h. A microplate reader (Bio-Rad, USA) was used to measure the absorbance values (OD) at 450 nm. All experiments were performed in triplicate.

CytoSelect 24-well cell invasion assay kits were used to perform transwell assays. First, 24-well plates were seeded with resuspended prostate cancer cells in serum-free medium. Then, the appropriate amount of cells and 200-μl medium were added to the upper layer of the compartment, with 700-μl medium containing 15% FBS added to the lower chamber. The cells were then cultured and incubated for 48 h. After 48 h, the noninvaded cells were wiped off and 4% paraformaldehyde was added for 1 h, followed by crystal violet staining for 1 h. The stain was washed under water until it no longer faded. The chamber was examined and images captured under a microscope.

### Experimental animals and xenograft models

This study was approved by the Ethics Committee of the Medical College of Xiamen University (Ethics No. XMULAC20200039). Four-week-old male BALB/c nude mice were acquired from Charles River in Beijing and kept in a pathogen-free environment. All animal experiments complied with the Xiamen University laboratory animal care and use guidelines. PC-3 wild-type and FTO knockout cells (1 × 10^7^ per mouse) were diluted in 75 ml of PBS and 75 ml of Matrigel (BD Biosciences) and subcutaneously implanted into immunodeficient male mice to investigate tumorigenesis. *V* = 1 / 2 (width^2^ × length) was used to compute the tumor volume. BALB/c nude mice were treated with LNCaP wild-type and TGF-β-treated cells (1 × 10^7^ per mouse) for the construction of a lung metastasis model. On the fourth day after cell injection, TGF-β (10 ng/ml) was injected intravenously every 4 d and PBS was administered to the control group. Mice were sacrificed after 5 weeks, and the metastatic lung tumors were examined using H&E and IHC.

### Statistical analysis

PFS and OS were calculated using the log-rank test, and survival curves were plotted for each dataset subgroups using the Kaplan–Meier plotter. For comparing continuous variables, one-way analysis of variance or an unpaired 2-tailed Student *t* test were used. ROC was used to find the best cutoff for continuous variables, and 2 groups were formed according to the cutoff for univariate logistic regression. An AUC of >0.5 was considered significant. GraphPad Prism 8 software (GraphPad Software, USA) was used to draw the plots (legend: *P* < 0.05 (*), *P* < 0.01 (**), and *P* < 0.001 (***)).

## Data Availability

All data obtained and/or analyzed in this study were available from the corresponding authors upon a reasonable request.
