# Supplementary material for: The FTO Mediated N6-Methyladenosine Modification of DDIT4 Regulation with Tumorigenesis and Metastasis in Prostate Cancer
Source: Research (Wash D C). 2024 Feb 21;7:0313. doi: 10.34133/research.0313 (PMC10879844; doi:10.34133/research.0313)
Supplement: Supplementary 1 — Figs. S1 to S7 Tables S1 to S3 [file research.0313.f1.doc]

**
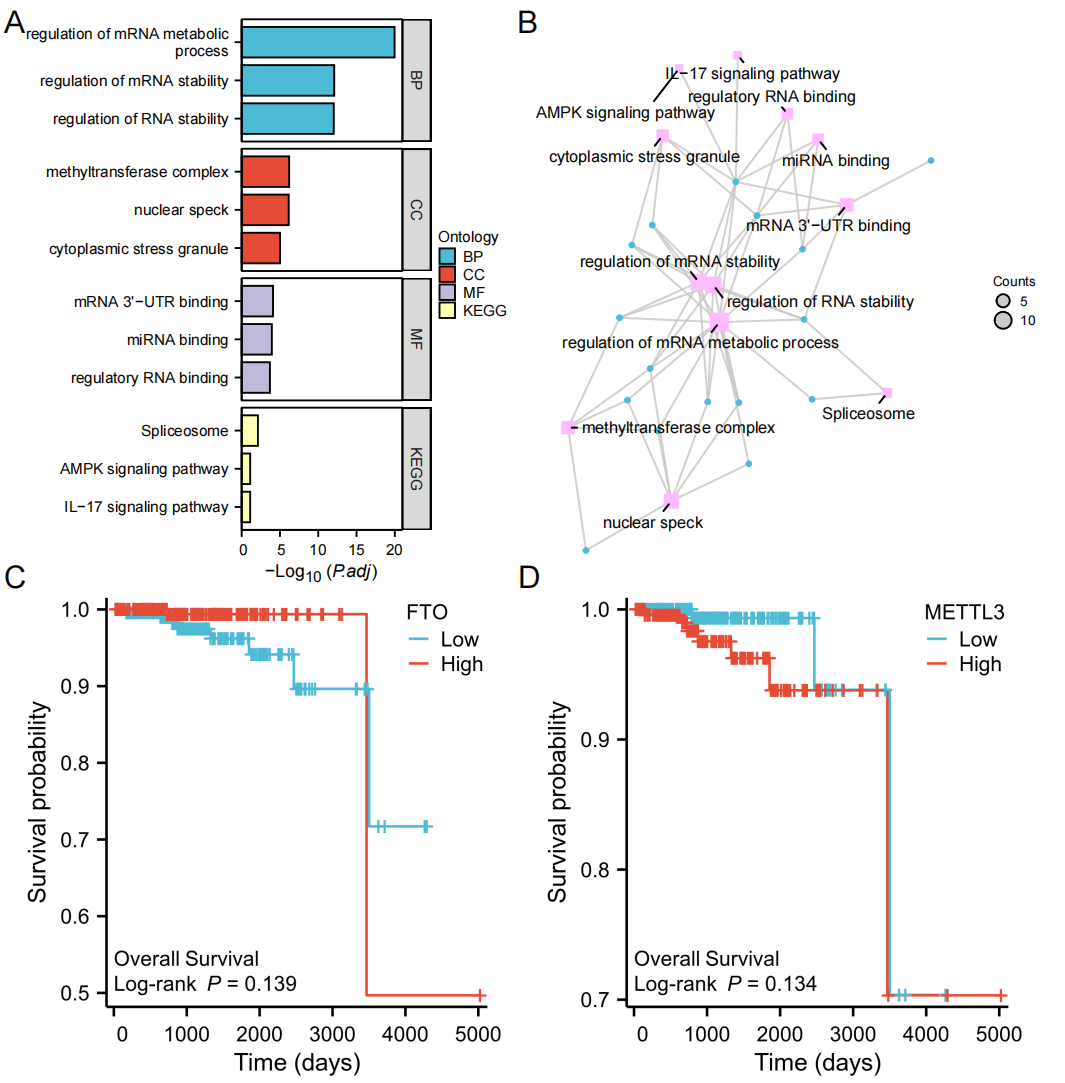
Figure S1: Enrichment and survival analyses of differentially expressed m6A regulators. (A)** GO enrichment analysis. **(B)** KEGG enrichment analysis. **(C)**Kaplan–Meier survival curves of OS based on FTO. **(D)** Kaplan–Meier survival curves of OS based on METTL3.


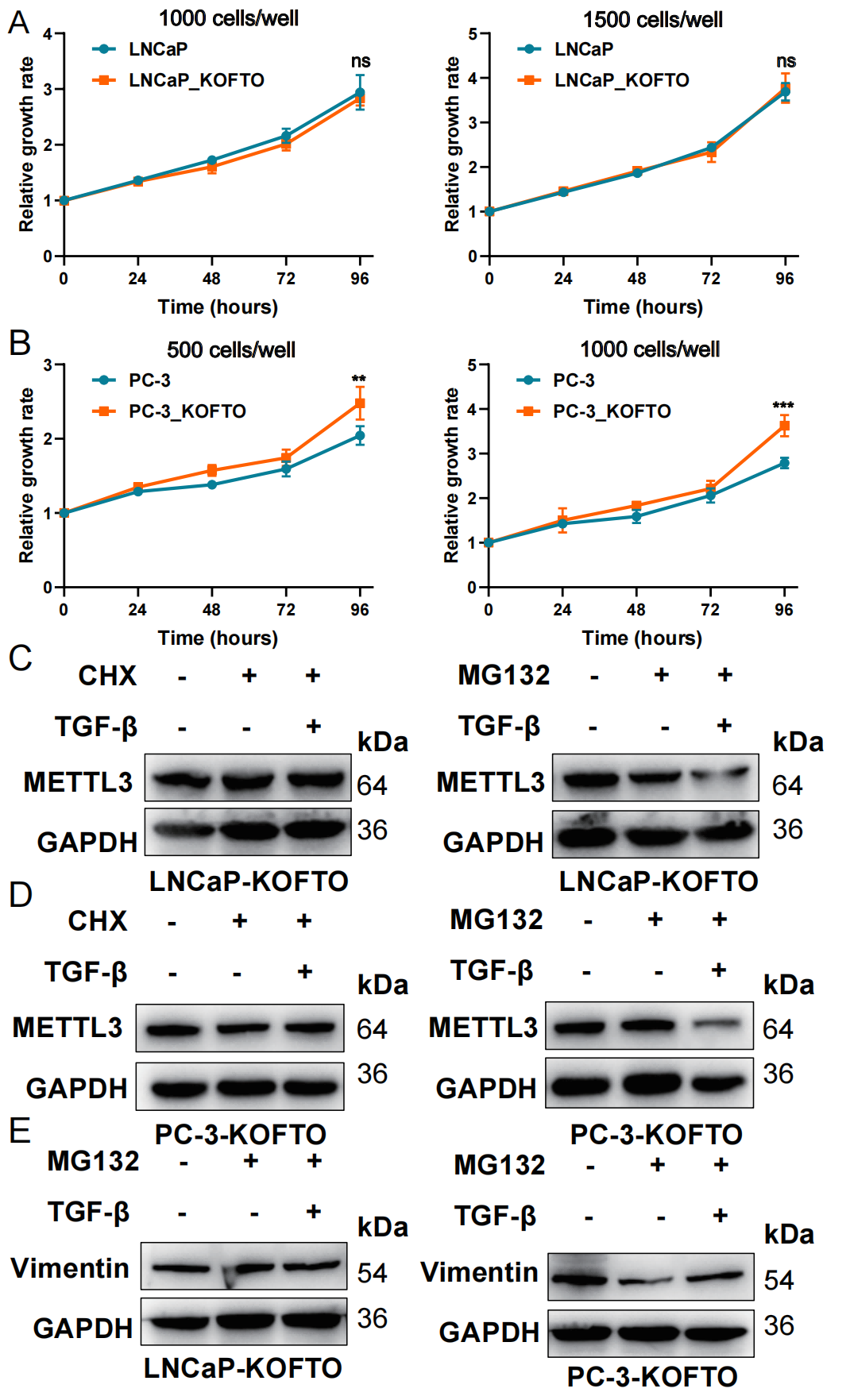


**Figure S2: Cell proliferation ability and immunohistochemical assays. (A)** Changes in the cell proliferation ability in LNCAP cells after FTO knockout. **(B)** Changes in the cell proliferation ability of PC-3 cells after FTO knockout. **(C, D)** FTO knockout cells were pretreated with CHX or MG-132 for 6 h and then further treated with or without 10 ng/mL TGF-β for 48 h. The expression of METTL3 was subsequently detected using western blot analysis. **(E)** FTO knockout cells were pretreated with MG-132 for 6 h and then further treated with or without 10 ng/mL TGF-β for 48 h. The expression of vimentin was subsequently detected using western blot analysis.


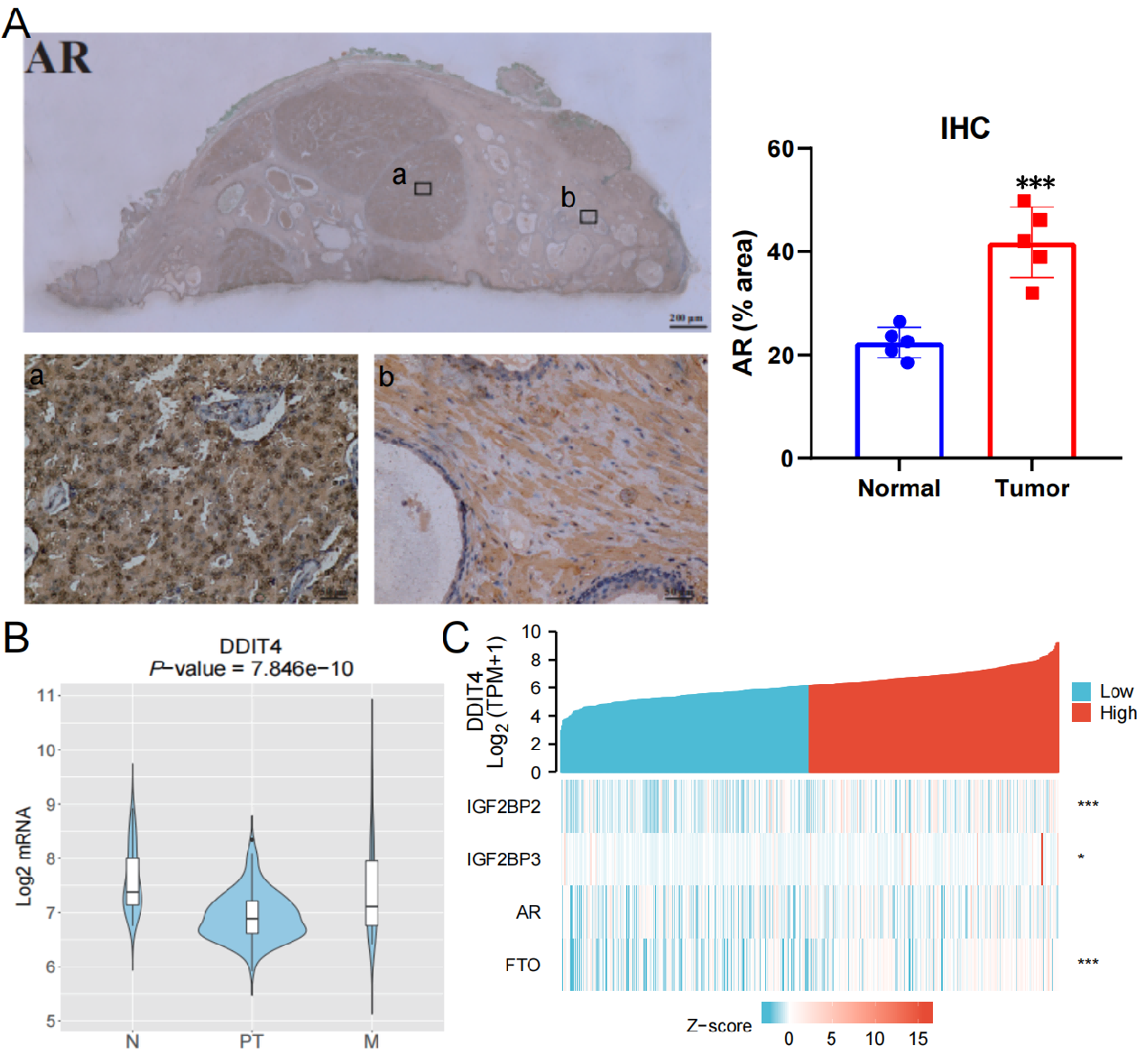


**Figure S3: Immunohistochemical analysis of DDIT4 expression in prostate cancer. (A)** IHC (AR)-stained paraffin-embedded sections obtained from patients with prostate cancer. Representative images of tumor tissues are shown in (a) and those of tumor adjacent tissues are shown in (b). **(B)** Expression levels of DDIT4 in normal tissues, primary tumors, and metastatic tissues. **(C)** Relationship between the high and low expression of DDIT4 and expression of IGF2BP2, IGF2BP3, AR, and FTO.


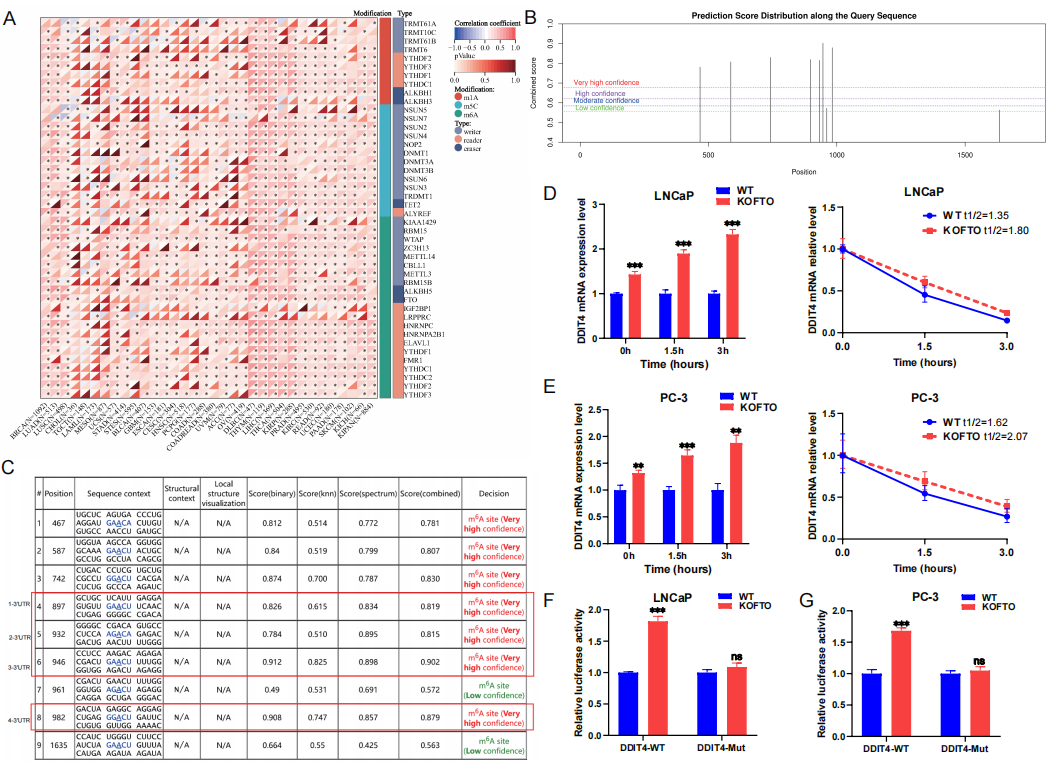


**Figure S4: Prediction of DDIT4 methylation modification sites. (A)** Pan-cancer methylation modification analysis of DDIT4. **(B)** m6A motif site of DDIT4 was analyzed using the SRAMP website. **(C)** Specific positions and prediction scores of the m6A motif sites in DDIT4. **(D)** mRNA half-lives were estimated after the indicated actinomycin D treatment in FTO knockout LNCaP and **(E)** PC-3 cells. **(F, G)** Relative luciferase level of wild-type DDIT4, but not of the mutated gene, was significantly increased in FTO knockout LNCaP and PC-3 cells.


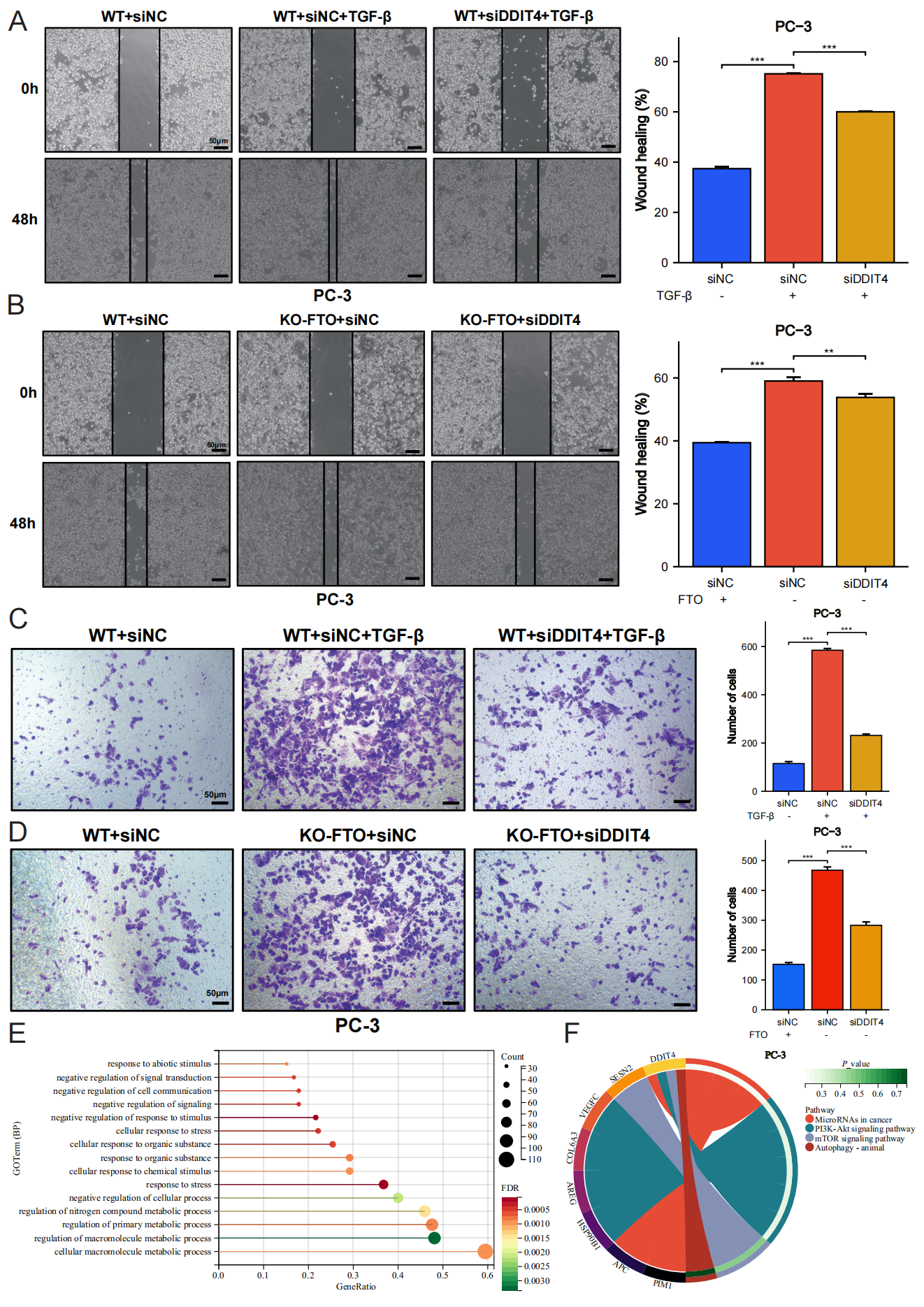


**Figure S5: Effect of DDIT4 expression level on cell biological characteristics in PC-3 cells. (A)** Wound-healing assays were performed to determine the effect of TGF-β and siDDIT4 and **(B)** that of FTO knockout and siDDIT4 on PC-3 cells. **(C)** Transwell assays were performed to determine the effect of TGF-β and siDDIT4 and **(D)** that of FTO knockout and siDDIT4 on PC-3 cells. **(E)** Top 15 BP terms, including DDIT4, enriched in PC-3 cells. **(F)** KEGG pathway genes, including DDIT4, enriched in PC-3 cells.


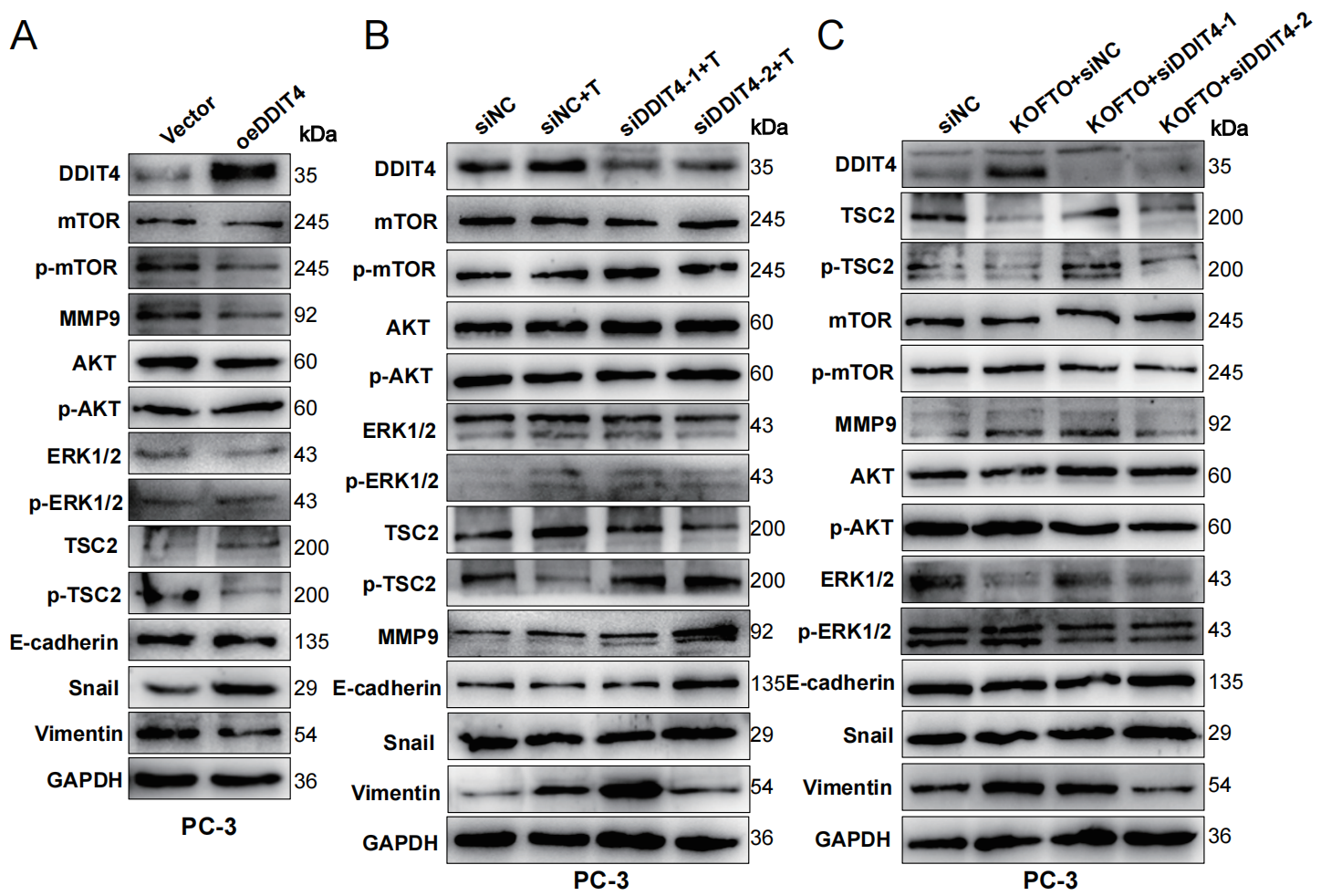


**Figure S6: Measurement of protein expression of genes in the PI3K-AKT-mTOR pathway in PC-3 cells using western blot analysis.** **(A)** Protein level of DDIT4 overexpression. **(B)** Protein expression of genes in cells after siDDIT4 and TGF-β treatment. **(C)** Protein expression of genes in FTO knockout cells after siDDIT4.

**
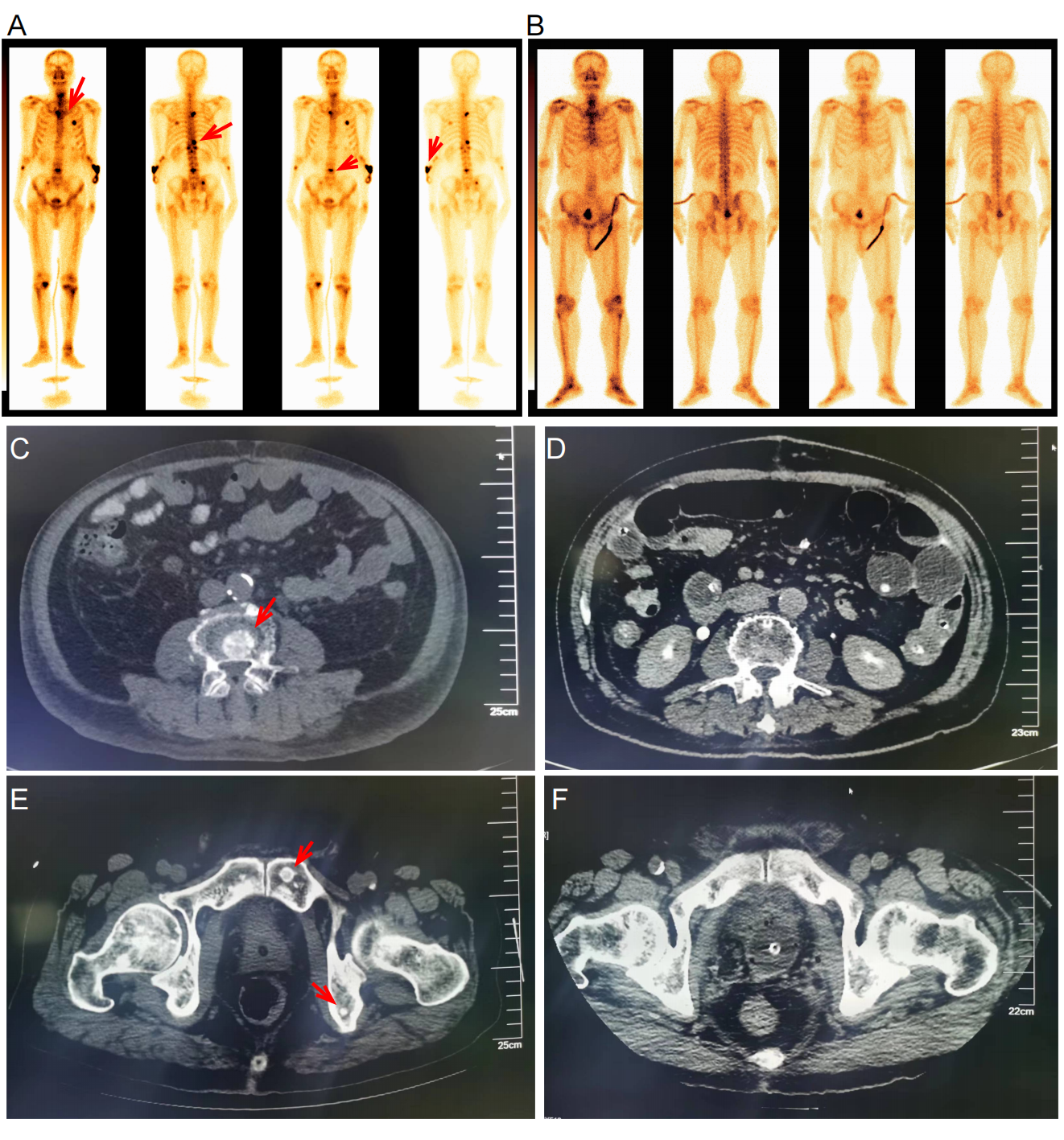
**

**Figure S7: Confirmation of bone metastasis in prostate cancer via imaging. (A)** Bone ECT was used to determine prostate cancer with and **(B)** without bone metastasis. **(C, D)** CT was used to determine prostate cancer with and **(D, F)** without bone metastasis.

**Table S1. qRT-PCR primer sequence**

| **Primer name** | **Primer sequence** |
| --- | --- |
| FTO-F (1) | GGCAGTGTACAGTTATAGCTGTG |
| FTO-R (1) | CCAAACCAGGTGTCTCTATGTC |
| METTL3-F | CTCTGCGGGAGAGGCTGCAG |
| METTL3-R | GAGAAGAAGTTGCTACACCAC |
| CDH1-F | GCCTCCTGAAAAGAGAGTGGAAG |
| CDH1-R | TGGCAGTGTCTCTCCAAATCCG |
| Vimentin-F | CGCCAACTACATCGACAAGGTGC |
| Vimentin-R | CTGGTCCACCTGCCGGCGCAG |
| DDIT4-F | AGGACGCACTTGTCTTAGCAG |
| DDIT4-R | GCAAGGACGAGGGCGAAGAG |
| IGF2BP2-F | ACCAGTGCAGAAGTCATCGT |
| IGF2BP2-R | GGAAGGGCTACATTCATCCGTT |
| IGF2BP3-F | GCTCATATCAGAGTGCCATCCT |
| IGF2BP3-R | TTTGGCATCTGCCTCTGTGGT |
| GAPDH-F | AGAAGGCTGGGGCTCATTTG |
| GAPDH-R | AGGGGCCATCCACAGTCTTC |
| FTO-F (2) | TAGCTCCAGACGGGAGCAG |
| FTO-R (2) | TTTGGGGGTCAGATAAGGG |

**Table S2. sgRNAand siRNA primer sequence**

| **Primer name** | **Primer sequence** |
| --- | --- |
| FTO-sgRNA1 | CTGAGAGAACTACATGCAGG |
| FTO-sgRNA2 | GAAGCGCACCCCGACTGCCG |
| si-FTO-1-sense | ACACUUGGCUCCCUUAUCUTT |
| si-FTO-1-antisense | AGAUAAGGGAGCCAAGUGUTT |
| si-FTO-2-sense | UGAAAUAUCCUAAACUAAUTT |
| siFTO-2-antisense | AUUAGUUUAGGAUAUUUCATT |
| si-IGF2BP2-sense | GAGAUAGAGAUUAUGAAGATT |
| si-IGF2BP2-antisense | UCUUCAUAAUCUCUAUCUCTT |
| si-IGF2BP3-sense | GGUGAAUGAACUUCAGAAUTT |
| si-IGF2BP3-antisense | AUUCUGAAGUUCAUUCACCTT |
| si-DDIT4-1-sense | GAUGAACACUUGUGUGCCATT |
| si-DDIT4-1-antisense | UGGCACACAAGUGUUCAUCTT |
| si-DDIT4-2-sense | GGAAUAGUGUUUCCCAGGATT |
| si-DDIT4-2-antisense | UCCUGGGAAACACUAUUCCTT |

**Table S3. m6A motif primer sequence**

| **Primer name** | **Primer sequence** |
| --- | --- |
| DDIT4-m6Asite1-F | GCCGGAGGAAGACACGG |
| DDIT4-m6Asite1-R | CTGCATCAGGTTGGCACACA |
| DDIT4-m6Asite2-F | CGGCTGGGCTCTCGACGCC |
| DDIT4-m6Asite2-R | CCGCACGGCTCGCTGTAGG |
| DDIT4-m6Asite3-F | CTCGACCCCAGCCTGGTGC |
| DDIT4-m6Asite3-R | AAGGGAGAGTTGGCGGAGC |
| DDIT4-m6Asite4,5-F | GGCTTCCGAGTCATCAAGAA |
| DDIT4-m6Asite4,5-R | CACCCCAAAAGTTCAGTCGT |
| DDIT4-m6Asite6,7-F | CCCTCCAAGACAGAGACGAC |
| DDIT4-m6Asite6,7-R | TTAGGTGGCTGCCTCAGTTT |
